# Understanding capacity fade in organic redox-flow batteries by combining spectroscopy with statistical inference techniques

**DOI:** 10.1038/s41467-023-39257-z

**Published:** 2023-06-16

**Authors:** Sanat Vibhas Modak, Wanggang Shen, Siddhant Singh, Dylan Herrera, Fairooz Oudeif, Bryan R. Goldsmith, Xun Huan, David G. Kwabi

**Affiliations:** 1 Department of Mechanical Engineering, University of Michigan, Ann Arbor, MI 48109 USA; 2 Department of Chemical Engineering, University of Michigan, Ann Arbor, MI 48109 USA; 3 Department of Chemistry, University of Michigan, Ann Arbor, MI 48109 USA

**Keywords:** Batteries, Batteries, Characterization and analytical techniques

## Abstract

Organic redox-active molecules are attractive as redox-flow battery (RFB) reactants because of their low anticipated costs and widely tunable properties. Unfortunately, many lab-scale flow cells experience rapid material degradation (from chemical and electrochemical decay mechanisms) and capacity fade during cycling (>0.1%/day) hindering their commercial deployment. In this work, we combine ultraviolet-visible spectrophotometry and statistical inference techniques to elucidate the Michael attack decay mechanism for 4,5-dihydroxy-1,3-benzenedisulfonic acid (BQDS), a once-promising positive electrolyte reactant for aqueous organic redox-flow batteries. We use Bayesian inference and multivariate curve resolution on the spectroscopic data to derive uncertainty-quantified reaction orders and rates for Michael attack, estimate the spectra of intermediate species and establish a quantitative connection between molecular decay and capacity fade. Our work illustrates the promise of using statistical inference to elucidate chemical and electrochemical mechanisms of capacity fade in organic redox-flow battery together with uncertainty quantification, in flow cell-based electrochemical systems.

## Abstract (web summary)

Organic redox-flow batteries have the potential to cheaply store renewable electricity at grid scale but require further development. Here, the authors show that combining spectroscopic measurements with statistical inference techniques can shed light on why these batteries lose capacity over time.

## Introduction

The development of low-cost grid-scale energy storage is necessary for widespread adoption of many renewable energy sources, given their intermittent nature. In this regard, redox-flow batteries (RFBs) are seen as a promising technology. An RFB comprises a pair of electrolyte reservoirs containing charge-storing redox-active materials which are separated by an ion-permeable membrane or separator. The electrolytes are pumped through a reactor cell, where they are oxidized and reduced cyclically as the RFB is charged and discharged^1^. This architecture imparts RFBs with the unique capability of independently scaling the energy storage capacity (which scales with the volume of electrolyte reservoirs and concentrations of charge-storing species) and power (which scales with the size of the reactor cell stack). As the energy-to-power ratio (or rated duration of discharge) increases, the levelized cost of stored energy asymptotes to the cost of the electrolytes^2,3^. For very cheap electrolytes, this cost may drop below that of standard enclosed (Li-ion) batteries^4,5^.

Aqueous-soluble organic and organometallic redox-active molecules have received considerable research attention as potential charge carriers in RFBs because their costs at scale are projected to be low^3,6^. Organic RFBs may thus store energy at lower levelized costs than state-of-the-art Li-ion systems if, in addition to low chemical cost, they also possess the right combination of solubility, chemical stability, and other electrochemical properties (e.g., redox potentials leading to high cell voltage and fast redox kinetics)^7^. Unfortunately, most aqueous organic flow cells experience temporal rates of capacity fade higher than 0.1%/day^8^, owing in large part to rapid chemical decomposition of their organic active materials. Such high fade rates render the vast majority of organic RFB chemistries unsuitable for practical deployment in RFB installations that are expected to last for decades.

Because redox-active organic molecules encompass a wide range of classes and are susceptible to a variety of decomposition mechanisms (e.g., nucleophilic attack, tautomerization, and hydrolysis)^8^, understanding how reactant conversion or decay leads to capacity fade is a critical but often difficult task. Such understanding often requires the deployment of new *operando* measurement tools^9–11^ and cycling protocols that allow the deconvolution of reactant decomposition from other sources of capacity fade^12–14^. For several candidate RFB charge carriers such as quinones^9,10,15^, iron-based organometallic complexes^16–18^ and nitrogen-containing aromatic molecules^19–21^, multiple hypotheses about the relationship between molecular decay and capacity fade have been advanced, some of which are mutually incompatible^8^. In other chemistries, such as one recently developed based on fluorenone^22^, there are complex equilibria among species in different redox and protonation states whose effect on capacity retention is not yet fully understood^23^. These challenges call for new techniques to discern the probabilities or relative contributions of various hypothesized mechanisms to capacity fade observed in flow cells. In particular, understanding and rigorously quantifying the degree to which data gathered from experiments confirm or challenge particular hypotheses about chemical versus electrochemical sources of capacity fade is critical for developing organic RFB chemistries.

Statistical learning of physical models and their parameters from experimental observations, broadly approached as an estimation or inference task, can be brought to bear on this issue. Estimation centers around the ideas of regression where the goal is to find the optimal value of parameters so that the model prediction best fits (explains) the observations (e.g., see ref. ^24^). However, these best fits are usually single-valued and do not quantify the uncertainty that is affected by, for example, the quantity and quality of observations. In contrast, inference seeks probabilistic solutions to convey the degree of uncertainty about different possible explanations that could have induced the observed data. Inference is typically carried out following the axioms of probability and Bayes’ theorem^25–27^, where an initial prior uncertainty distribution is appropriately updated to a posterior uncertainty distribution given the newly acquired observations. The Bayesian update rule naturally incorporates new data that may materialize sequentially over time and offers a coherent representation of evidence aggregation. Bayesian inference is also advantageous for accommodating sparse, noisy, and indirect measurements, consolidating data sets from different sources and of varying quality, and permitting the injection of domain knowledge and expert opinion to the learning process^28^. Beyond parameter inference, the Bayesian framework also extends to model selection^29,30^, allowing one to compare various “packages” of hypotheses and assumptions manifesting as different model structures and parameterizations (e.g., different reaction mechanisms).

Bayesian inference and related probabilistic techniques have been applied to several problems in electrocatalysis and battery science, including failure prediction^31^ and the development of lifetime-extending charging protocols in Li-ion batteries^32^, analyte labeling^33^, model/variable selection and parameter estimation for Li-ion battery electrodes^34^, electrochemical cell design^35,36^, Tafel slope interpretation,^37^, and materials discovery^38,39^. However, no studies of which we are aware have applied these techniques to understand capacity fade or reactant decay in organic RFBs, nor has Bayesian model selection been deployed in these contexts.

In this work, we apply Bayesian inference and multivariate curve resolution-alternating least squares (MCR-ALS) methods to spectroscopic analysis of the decomposition of oxidized 4,5-dihydroxy-1,3-benzenedisulfonic acid (BQDS) or Tiron, an ortho-hydroquinone derivative previously investigated as a positive electrolyte material in aqueous RFBs^40–42^. Whereas Bayesian inference applies broadly, MCR-ALS is specifically suited to spectrophotometry, as it uses iterative optimization under well-defined physical constraints for resolving mixed signals in a multi-component system into its pure components^43^. MCR-ALS is applicable to understanding speciation in multi-component chemical systems via optical absorbance, where absorbance at a given wavelength might be linearly proportional to the concentrations of each component. Previous work has shown that oxidized BQDS is susceptible to a self-discharge reaction with water known as Michael addition/attack^40–42,44^, which results in the formation of a series of hydroxyl-substituted para-hydroquinone species with lower redox potentials than BQDS. However, the intrinsic rates of Michael addition, and whether these rates are modified under *operando* cycling conditions, are not known. This information is critical for establishing a quantitative link between reactant decay/conversion and capacity fade.

**Fig. 1:**
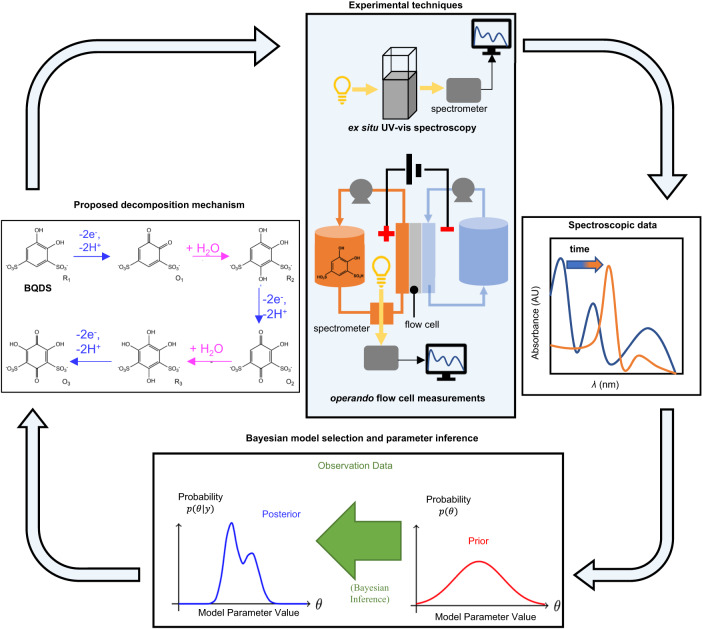
Graphical summary of the present work. We applied Bayesian model selection and inference, as well as multivariate curve resolution techniques, to spectroscopic data obtained ex situ and in *operando* flow cells in order to elucidate and quantify the kinetics of Michael attack of BQDS.

The main contributions of our article are schematized in Fig. 1 and are as follows: 1. We performed Bayesian model selection to identify the most plausible kinetic scheme for the decay of BQDS based on ultraviolet-visible (UV–vis) spectrophotometry of a sacrificial oxidant, which acted as a reporter of Michael attack; 2. By applying Bayesian parameter inference and MCR-ALS to the UV–vis data, we obtained uncertainty-quantified estimates of the rates of Michael attack of BQDS both ex situ and inside an operating flow cell; and 3. We individually isolated the UV–vis spectra of all oxidation and Michael attack products by applying MCR-ALS to spectroscopic data obtained from the *operando* BQDS-containing flow cell.

Our paper is structured as follows. Section “Spectroscopic measurement of michael attack of BQDS” reports experimental details and measurements of Michael attack of BQDS ex situ via UV–vis spectrophotometry of the concentration of the sacrificial oxidant. Section “Model selection and uncertainty-quantified rates of Michael attack using Bayesian inference and multivariate curve resolution analysis” describes our application to the UV–vis data of Bayesian model selection and inference of the relevant decay rate constants. Section “Spectrophotometric analysis of BQDS decomposition in an operando flow cell” reports experimental details regarding BQDS oxidation and Michael attack under electrochemical cycling. In Section “Estimation of decay rate constants and UV–vis spectra of oxidation products”, we apply MCR-ALS to the *operando* UV–vis data, extracting the relevant decay rate constants as well as spectra for each oxidation/intermediate product. Finally, in Section “Discussion” we corroborate our results using nuclear magnetic resonance (NMR) analysis and density functional theory (DFT) calculations of the reaction energetics for Michael addition to BQDS. Section “Methods” reports all experimental and computational methods. Our work demonstrates the promise of using statistical inference techniques to elucidate and distinguish between chemical and electrochemical mechanisms of capacity fade in organic RFBs, and, more generally, for understanding variable-timescale molecular transformations in other flow cell-based electrochemical applications.

## Results and discussion

### Spectroscopic measurement of michael attack of BQDS

We first examined Michael attack of BQDS in 1 M H_2_SO_4_ via spectrophotometric tracking of the concentration of a sacrificial chemical oxidant. BQDS is attractive as a positive electrolyte material in aqueous organic RFBs because of its high solubility (4 M)^40^ and redox potential (0.65 V vs Ag/AgCl, see cyclic voltammetry measurement in Supplementary Fig. 1). However, previous cell cycling experiments^40,42^ have shown that oxidized BQDS is susceptible to self discharge—this results in a progressively lower energy efficiency and the development of a Coulombic imbalance between the negative and positive electrolytes. Based on ex situ voltammetry and chemical analysis, it has been proposed that the origin of self discharge is one^42^ or two^40^ spontaneous nucleophilic additions of water to oxidized BQDS and its successive oxidized derivatives(denoted O_1_, O_2_, and O_3_, respectively), which results in the formation of reduced, hydroxyl-substituted hydroquinone species (R_1_, R_2_ and R_3_), as illustrated in Fig. 1. Note that self discharge of BQDS does not entail oxygen or hydrogen evolution from water. Rather, the Michael addition reaction entails the formation of an adduct between water and a quinone derivative, followed by electron transfer within the adduct, and proton transfer between the adduct and solution. Because the overall result is that ketone groups in the quinone are reduced and protonated, the process is akin to discharge (reduction) of the quinone. Because oxidized BQDS and its successive oxidized derivatives (Fig. 1) are also susceptible to dimerization and other cyclization reactions with each corresponding reduced form^44^, it is necessary to maintain each redox couple in the oxidized form in order to isolate the rates of conversion of O_1_ to O_2_, O_2_ to O_3_, etc., hereafter denoted as *k*_1_ and *k*_2_, etc. We therefore sought a sacrificial oxidant that would maintain the oxidized form of each redox couple, and then to spectrophotometrically track the decrease in its concentration over time, the rate of which would correspond to the rate of Michael addition. The ideal oxidant must have (1) a redox potential that is high enough to oxidize BQDS but not to oxidize water, and (2) optical absorbance features that do not coincide with, or are negligible compared with those of BQDS. Both of these criteria are met by potassium dichromate (K_2_Cr_2_O_7_), which has a redox potential of 1.3 V vs SHE (Supplementary Fig. 1), and whose UV–vis spectrum exhibits prominent peaks at 350 and 445 nm (Supplementary Fig. 2a, b), where absorbance of BQDS is near zero. Two-electron oxidation of BQDS or any other reduced hydroquinone derivatives by one mole of dichromate is expected to proceed according to the reaction:

1 $${{{{{{{{\rm{Cr}}}}}}}}}_{2}{{{{{{{{{\rm{O}}}}}}}}}_{7}}^{2-}+{3{{{{{{{{\rm{R}}}}}}}}}_{x}}^{2-}-+14{{{{{{{{\rm{H}}}}}}}}}^{+}\to 2{{{{{{{{\rm{Cr}}}}}}}}}^{3+}+3{{{{{{{{\rm{O}}}}}}}}}_{x}+7{{{{{{{{\rm{H}}}}}}}}}_{2}{{{{{{{\rm{O}}}}}}}}$$

where O_*x*_ and R_*x*_ represent oxidized and reduced derivatives, respectively.

**Fig. 2:**
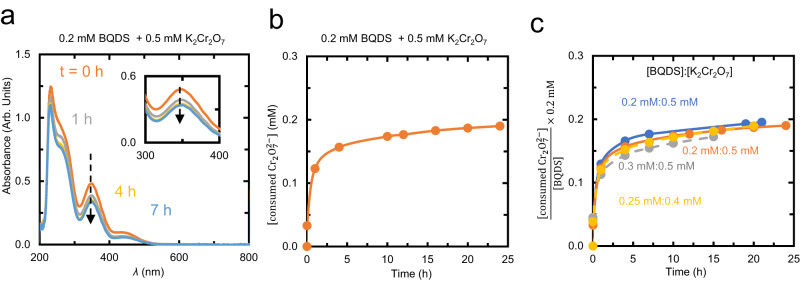
Tracking the progress of Michael addition using K_2_Cr_2_O_7_ as a sacrificial oxidant. **a** UV–vis spectra over time of a mixture of 0.2 mM BQDS and 0.5 mM K_2_Cr_2_O_7_ in 1 M H_2_SO_4_. The decrease in the absorbance of the peak at 350 nm with time is highlighted in the inset. **b** Estimate of the concentration of K_2_Cr_2_O_7_ consumed based on the peak intensity at 350 nm. **c** Estimates for the concentration of K_2_Cr_2_O_7_ consumed for all experiments run with other concentrations of BQDS and K_2_Cr_2_O_7_ normalized to an initial BQDS concentration of 0.2 mM.

The reduction in [K_2_Cr_2_O_7_] in the presence of oxidized BQDS was consistent with two successive Michael additions. Figure 2a shows selected UV–vis spectra of 0.5 mM K_2_Cr_2_O_7_ after the addition of 0.2 mM BQDS. The intensity of the spectra decreased uniformly over the course of 20 h, consistent with a decrease in [K_2_Cr_2_O_7_]. Because K_2_Cr_2_O_7_ was added in stoichiometric excess to BQDS, we tracked the decrease in the absorbance peak at 350 nm as a reporter of [K_2_Cr_2_O_7_], based on a calibration curve (Supplementary Fig. 2c), and thus the reaction depicted in Eq. (1) (Fig. 2b). Upon the addition of the BQDS, there was an instantaneous 0.07 mM decrease in [K_2_Cr_2_O_7_] that was commensurate with two-electron oxidation of BQDS to its oxidized form O_1_ (i.e., R_1_ to O_1_). Over the next hour, the rate of consumption of K_2_Cr_2_O_7_ proceeded at an average of about 0.08 mM/h, before slowing to 0.001 mM/h over the subsequent 21 h. The total consumed [K_2_Cr_2_O_7_] appeared to asymptotically approach 0.2 mM. This total consumption is consistent with three discrete two-electron oxidation events: one for conversion of R_1_ to O_1_, and one each for R_2_ to O_2_ and R_3_ to O_3_.

Control experiments confirmed that both BQDS and K_2_Cr_2_O_7_ were required for the observed spectral changes. No spectral changes were observed for BQDS in the absence of K_2_Cr_2_O_7_ (Supplementary Fig. 3)—consistent with water oxidation being insignificant—and vice versa (Supplementary Fig. 1). The control experiments also confirm the absence of any lamp drift because there is no shift observed in the zero-absorbance baseline above 500 nm over time as can be seen in Fig. 2a. We have also shown the lamp spectrum in Supplementary Fig. 4, where no observable drift is seen. Oxidation of BQDS in a thin-layer spectroelectrochemical cell for 19 h also revealed, consistent with previous work^40,42^, the disappearance of the original BQDS redox feature, and the emergence of a new redox pair ~ 200 mV lower, around 0.65 V vs SHE (Supplementary Fig. 1). This new redox pair has been attributed to O_3_^40^ as, being a para-quinone, it is expected to have a lower reduction potential than the ortho-quinone O_1_^45^.

We conducted three more BQDS oxidation measurements with different initial BQDS concentrations (Fig. 2c), and initial [K_2_Cr_2_O_7_]:[BQDS] ratios varying between 1.6 and 2.5—but always in excess of 1, which is the stoichiometric ratio required for three two-electron oxidation reactions based on Eq. (1). Supplementary Fig. 5 reports the [K_2_Cr_2_O_7_] consumed over time for these measurements. Each trace asymptotes close to the expected steady state. When normalized to the same BQDS concentration (Fig. 2c), the traces overlap, strongly suggesting that the chemical decay of BQDS is first order in [O_*x*_], in agreement with the decomposition scheme in Fig. 1.

### Model selection and uncertainty-quantified rates of Michael attack using Bayesian inference and multivariate curve resolution analysis

Taken together, the spectroscopic data above constitute prima facie evidence that oxidized BQDS and its subsequent derivatives are susceptible to two, first-order Michael additions. We therefore sought to rigorously quantify the probability that the above data are explained by the model in Fig. 1, and then to calculate uncertainty-quantified reaction orders and rates for each individual Michael addition.

We first evaluated the most likely number of Michael addition reactions indicated by the above spectroscopic data, among one (i.e., conversion of O_1_ to O_2_), two (O_1_ to O_2_ to O_3_) and three (O_1_ to O_2_ to O_3_ to O_4_). The unknown model parameters include reaction rates and orders for each individual transition (e.g., *k*_1_, *k*_2_, *m*_1_ and *m*_2_ for the model with two Michael additions). We chose a prior probability density function (PDF) of the reaction rate to be uniformly distributed over four orders of magnitude in log space, i.e., ${\log }_{10}k \sim {{{{{{{\mathcal{U}}}}}}}}[-6,-2]$ for all *k* terms, and a reaction order *m* that could take a value from {0, 1, 2} with uniform probability, i.e., ${\mathbb{P}}(m=0)={\mathbb{P}}(m=1)={\mathbb{P}}(m=2)=\frac{1}{3}$). We used the peak in the optical absorbance spectrum at 350 nm as our observation of [K_2_Cr_2_O_7_] because the optical absorbance from K_2_Cr_2_O_7_ at that wavelength is significantly stronger than that of BQDS (Supplementary Fig. 2a), its derivatives^42^, and Cr^3+^. Moreover, we assumed that the noise on absorbance of different snapshots is independent and identically distributed, such that the covariance matrix Σ_*ϵ*_ of the additive Gaussian error *ϵ* in Eq. (8) is a diagonal matrix with identical entries. The scale of the additive noise was determined by the standard deviation of the 350 nm peak from calibration curve measurements (Supplementary Fig. 2b). As shown in Supplementary Fig. 6, the standard deviation is about 0.0096 at 350 nm, therefore, we set Σ_*ϵ*_’s diagonal entries to 0.0096^2^.

**Fig. 3:**
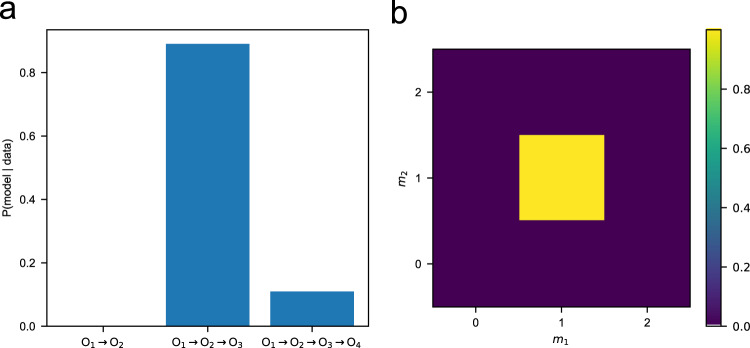
Bayesian inference predictions for probabilities of competing models. **a** Results of Bayesian model selection, showing the probabilities that UV–vis data in Fig. 2 are explained by a decay model with one versus two versus three Michael addition reactions. The results indicate that two Michael additions has a significantly higher probability compared with the other models. **b** Marginal posterior probability distribution of the reaction orders (*m*) for the BQDS decay model with two Michael addition reactions (${\mathbb{P}}({m}_{1},{m}_{2}|y,{{{{{{{\mathcal{M}}}}}}}})$). The combination *m*_1_ = *m*_2_ = 1 has a probability very close to 1.

Figure 3a shows the results of Bayesian model selection. The model posterior probabilities were computed following Eq. (10), which comprises random sampling of rate constants and reaction orders from their prior distribution and then comparing their predictions of K_2_Cr_2_O_7_ consumption with corresponding experimental values from UV–vis spectra. The Michael addition reaction was solved numerically using a 4th order Runge–Kutta time-marching algorithm, to ensure good numerical accuracy. We found using *N* = 10^6^ samples in Eq. (10) that the model with two Michael additions (i.e., conversion of O_1_ to O_2_ and then O_3_) has the highest model evidence among three candidate models. This result is consistent with the stoichiometry of K_2_Cr_2_O_7_ consumption in Section “Spectroscopic measurement of michael attack of BQDS”.

We then applied Bayesian inference to ascertain unknown parameters for the most plausible model. We first determined the most probable *m* values by computing ${\mathbb{P}}({m}_{1},\,{m}_{2}|y,\,{{{{{{{\mathcal{M}}}}}}}})$, then sampled *k* while the reaction orders were fixed to their most probable values. This strategy was chosen instead of sampling *k* and *m* jointly because sampling from a mixed continuous-discrete space is computationally expensive, and there are only nine possible combinations of reaction order terms for the two-Michael addition model. Figure 3b shows the marginal posterior of *m*_1_ and *m*_2_ given the UV–vis data, which was computed through:

2 $${\mathbb{P}}({m}_{1},\, {m}_{2}|y,\, {{{{{{{\mathcal{M}}}}}}}})	=\int\,p({k}_{1},\, {k}_{2},\, {m}_{1},\, {m}_{2}|y,\, {{{{{{{\mathcal{M}}}}}}}})\,d{k}_{1}\,d{k}_{2}\\ 	 \propto {\mathbb{P}}({m}_{1},\,{m}_{2}|{{{{{{{\mathcal{M}}}}}}}})\int\,p(y|{k}_{1},\, {k}_{2},\, {m}_{1},\, {m}_{2},\, {{{{{{{\mathcal{M}}}}}}}})\,p({k}_{1},\, {k}_{2}|{{{{{{{\mathcal{M}}}}}}}})\,d{k}_{1}\,d{k}_{2},$$

where the integral was estimated by taking the average of likelihood values with *k*_1_ and *k*_2_ sampled from the prior. Our results revealed that the combination *m*_1_ = *m*_2_ = 1—that is, conversion of O_1_ to O_2_ and O_2_ to O_3_ both being first order in [O_*x*_]—is the most likely, with a posterior probability close to 1. Importantly, our analysis rules out the rather unlikely possibility that the apparently first-order reactivity inferred in Section “Spectroscopic measurement of michael attack of BQDS” was due to one reaction being zeroth order and the other being second order in [O_*x*_].

**Fig. 4:**
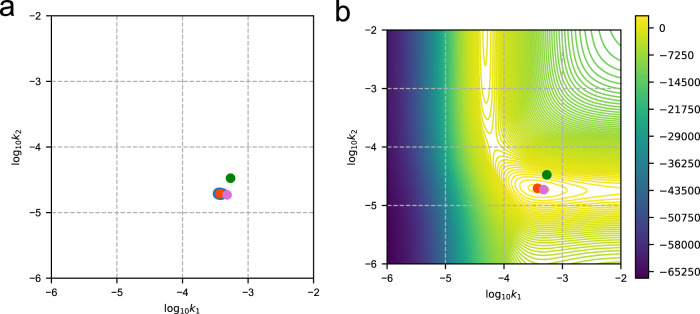
Bayesian inference predictions of the Michael addition reaction constants. **a** Bayesian inference of the posterior distribution for *k*_1_ and *k*_2_. **b** Contour plot of the logarithm of the un-normalized posterior PDF (i.e., of the numerator in Bayes' rule Eq. (7)) for ${\log }_{10}{k}_{1}$ and ${\log }_{10}{k}_{2}$ at the most probable reaction orders *m*_1_ = *m*_2_ = 1. Blue points (cluster) are the MCMC samples, and the red point marks the sample with the highest posterior PDF located at *k*_1_ = 3.768 × 10^−4^ and *k*_2_ = 1.986 × 10^−5^ s^−1^. The green point marks the location of *k*_1_ and *k*_2_ obtained by applying MCR-ALS to the spectroscopic data corresponding to BQDS decay from K_2_Cr_2_O_7_ consumption, whereas the purple point marks the location of *k*_1_ and *k*_2_ obtained by applying MCR-ALS to BQDS decay in *operando*.

We next estimated the rate constants for each Michael addition using Bayesian parameter inference. Figure 4a plots samples generated from the posterior for reaction rates $p({\log }_{10}{k}_{1},\,{\log }_{10}{k}_{2}|y,\,{m}_{1}=1,\,{m}_{2}=1,\,{{{{{{{\mathcal{M}}}}}}}})$ and Fig. 4b reports the logarithm of the un-normalized posterior PDF (i.e., of the numerator in Bayes’ rule Eq. (7)). Our results indicate that there are two local maxima in the contour plot, but the bottom right local maxima has a much higher probability density value, and consequently only one high probability cluster emerged from the MCMC samples. The bottom right cluster corresponds to *k*_1_ = 3.768 × 10^−4^ and *k*_2_ = 1.986 × 10^−5^ s^−1^. The standard deviations of samples are 1.248 × 10^−5^ and 2.684 × 10^−7^ s^−1^ for *k*_1_ and *k*_2_, respectively. These results indicate that Michael attack to O_1_ is considerably more rapid than to O_2_, with half lives of conversion of 0.51 and 9.69 h, respectively.

**Fig. 5:**
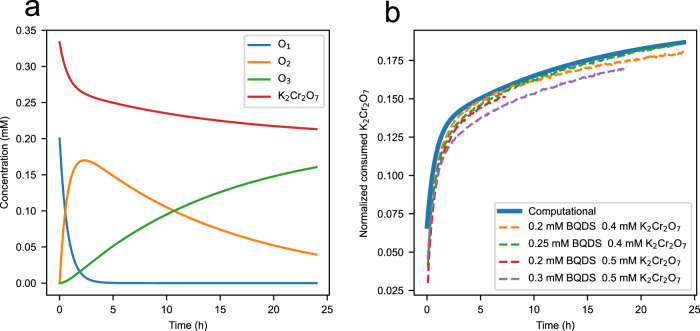
Bayesian inference predictions of BQDS derivative concentrations. **a** Computational evolution of oxidized species for the case with 0.4 mM initial [K_2_Cr_2_O_7_] and 0.2 mM initial [BQDS] assuming Bayesian-inferred rate constants, and **b** Computational and experimental estimates of consumption of K_2_Cr_2_O_7_ over time, where the consumed K_2_Cr_2_O_7_ concentration is normalized by dividing it by the initial BQDS concentration and multiplying by 0.2 mM.

Figure 5a shows the expected temporal evolution in the concentration of all oxidized species for initial concentrations of 0.4 and 0.2 mM for K_2_Cr_2_O_7_ and BQDS, respectively, reported in Fig. 2c, assuming first-order kinetics for Michael attack and *k*_1_ and *k*_2_ values set at the highest posterior probability (red point) from Fig. 4a. The temporal evolution of the same species in the other experiments with other initial K_2_Cr_2_O_7_ and BQDS concentrations are shown in Supplementary Fig. 7. Figure 5b reports the computational temporal consumption of dichromate, normalized to an initial concentration of 0.2 mM and overlaid with similarly normalized experimental data in Fig. 2c. There is excellent agreement between the computational and experimental transients in [K_2_Cr_2_O_7_], which strongly supports the validity of our Bayesian inference results.

In addition to Bayesian inference, we also estimated the decay rate constants by applying the MCR-ALS suite of applications to the spectroscopic data. Given the first-order reactivity of O_1_ and O_2_ determined above, the decay of BQDS and its oxidized derivatives can be modelled as a first-order multi-component kinetics problem, as follows:

3 $$\frac{d[{{{{{{{{\rm{O}}}}}}}}}_{1}]}{dt}=-{k}_{1}[{{{{{{{{\rm{O}}}}}}}}}_{1}]$$

4 $$\frac{d[{{{{{{{{\rm{O}}}}}}}}}_{2}]}{dt}={k}_{1}[{{{{{{{{\rm{O}}}}}}}}}_{1}]-{k}_{2}[{{{{{{{{\rm{O}}}}}}}}}_{2}]$$

5 $$\frac{d[{{{{{{{{\rm{O}}}}}}}}}_{3}]}{dt}={k}_{2}[{{{{{{{{\rm{O}}}}}}}}}_{2}]$$

where we have neglected the rapid chemical oxidation reactions. This system of equations has an analytical solution, shown in Supplementary Eqs. (1)–(3) for [O_1_], [O_2_] and [O_3_] respectively in the Supplementary Information.

The concentration of consumed K_2_Cr_2_O_7_ is a function of the instantaneous concentrations of O_2_ and O_3_:

6 $${[{{{{{{{{\rm{K}}}}}}}}}_{2}{{{{{{{{\rm{Cr}}}}}}}}}_{2}{{{{{{{{\rm{O}}}}}}}}}_{7}]}_{{{{{{{{\rm{consumed}}}}}}}}}=\frac{1}{3}[{{{{{{{{\rm{O}}}}}}}}}_{2}]+\frac{2}{3}[{{{{{{{{\rm{O}}}}}}}}}_{3}].$$

By substituting the analytical expressions for O_2_ and O_3_ into Eq. (6), the consumed [K_2_Cr_2_O_7_] can be written as a function of *k*_1_ and *k*_2_. Thus, knowing the concentration of K_2_Cr_2_O_7_ at each time step permits one to determine the values of *k*_1_ and *k*_2_. We approached this problem by first calculating [K_2_Cr_2_O_7_] from each UV–vis spectrum based on the peak at 350 nm. This was done using MCR-ALS techniques (Supplementary Section 2). The values for *k*_1_ and *k*_2_ were then determined following Eq. (6) by using the concentrations determined for consumed [K_2_Cr_2_O_7_] using MCR-ALS coupled with a fitting procedure from MATLAB’s curve fitting toolbox^46^. The results obtained for *k*_1_ and *k*_2_ for the four data sets with different initial concentrations are presented in Table 1. The values for *k*_1_ and *k*_2_ averaged over these four data sets are 5.40 × 10^−4^ s^−1^ and 3.34 × 10^−4^ s^−1^, which are reasonably consistent with the decay rate constants calculated from Bayesian inference (green points in Fig. 4).

**Table 1:** Michael attack rate constants determined by fitting first-order kinetics to consumed K_2_Cr_2_O_7_ concentration from UV–vis data

[K_2_Cr_2_O_7_]_0_(mM)	[BQDS]_0_(mM)	*k*_1_(10^−4^*s*^−1^)	${\sigma }_{{k}_{1}}(1{0}^{-5}{s}^{-1})$	*k*_2_(10^−5^*s*^−1^)	${\sigma }_{{k}_{2}}(1{0}^{-7}{s}^{-1})$
0.40	0.20	6.52	2.53	3.11	3.14
0.40	0.25	5.27	0.66	2.95	1.09
0.50	0.20	6.04	1.09	5.39	5.89
0.50	0.30	3.79	0.24	1.90	0.46
Average, MCR-ALS		5.40		3.34	
Bayesian inference		3.77	1.25	1.99	2.68

Rate constants obtained using Bayesian inference are shown for comparison.

### Spectrophotometric analysis of BQDS decomposition in an *operando* flow cell

The preceding spectroscopic measurements and analysis were aimed at quantifying the rate(s) of purely chemical decomposition of BQDS, i.e., in the absence of an applied potential or interaction with typical flow cell hardware (porous electrodes and membrane). To evaluate electrochemical BQDS decomposition during cycling of a flow cell, we set up a compositionally asymmetric flow cell whose capacity-limiting electrolyte (CLE) comprised 5–15 mM of BQDS in a supporting electrolyte of 0.9 M H_2_SO_4_ and 0.1 M CH_3_COOH. An excess of Methylene Blue was used as the counter electrolyte, and the two electrolytes were separated by a Nafion 115 membrane. The CLE was circulated among the electrolyte reservoir, cell, and an absorbance micro cross flow cell with a path length of 0.15 mm, for continuous UV–vis measurements (Supplementary Fig. 14). No K_2_Cr_2_O_7_ was present in the CLE, meaning that all UV–vis spectra represented a concentration-weighted average of BQDS and all its oxidation/decay products.

**Fig. 6:**
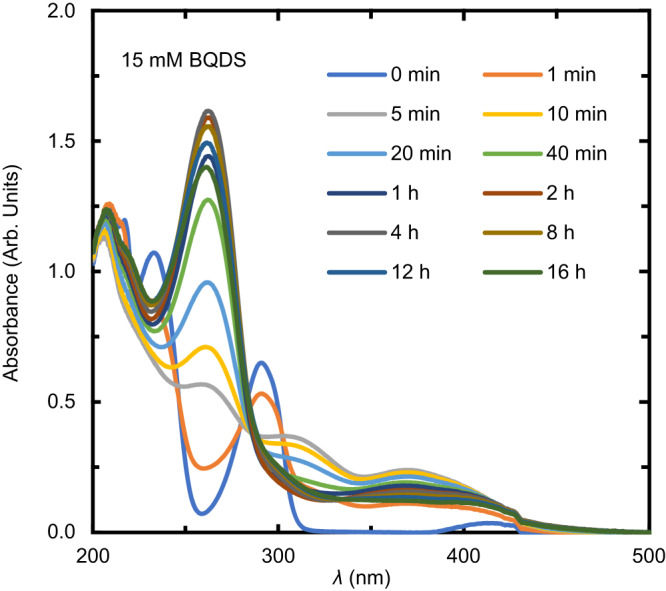
Operando UV-vis measurements. Evolution of the *operando* UV–vis spectrum of an electrolyte initially containing 15 mM BQDS during continuous oxidation in a BQDS-MB flow cell.

Figure 6 reports *operando* UV–vis spectra of a CLE containing 15 mM of BQDS after potentiostatic oxidation at a cell voltage of 0.8 V. Over the course of the experiment, the absorbance peak at 305 nm in the R_1_ spectrum diminished, while a new peak at 260 nm evolved, increased in intensity for roughly 2 h, and then slowly reduced in intensity. A similar trend has been observed previously^42^.

We carried out two other experiments at BQDS concentrations of 5 and 8 mM but under otherwise identical potentiostatic conditions (Supplementary Fig. 8a and b, respectively); Supplementary Fig. 8c reports the current response for all three cells and Supplementary Fig. 8d shows the peak intensities at 260 and 305 nm coinciding when the UV–vis spectra are scaled to a concentration of 5 mM. This concentration-independent scaling is consistent with first-order kinetics for all species conversions, as has been demonstrated in the preceding sections.

We verified that these spectral trends did not result from some spurious chemical interaction between BQDS and the hardware within the absorbance cross flow cell. To do so, we set up an identical flow cell without the cross flow cell apparatus, and periodically took aliquots from the CLE for in situ UV–vis analysis. Supplementary Fig. 8e reports the absorbance at 260 nm over time for two such in situ measurements; they show clear qualitative agreement with the UV–vis trends for the *operando* setup in Supplementary Fig. 8d.

For the practical application of this method to other molecules and mechanisms, certain constraints and limitations have to be considered. First, the UV–vis spectra of the reactants and the degradation products need to be intense enough to be detected and significantly distinct from one another. Second, a detectable amount of the degradation products must form during the experiment. To give an example, assuming a change in absorbance of at least 0.05 is required to detect a decay product whose molar absorptivity is 10^−3^ M^−1^cm^−1^, over a cycling period of no more than 10 days, the molecular decay/capacity fade rate should be at least 0.5%/day. For timescales larger than 10 days, the in situ method can still be used, where aliquots can be drawn every few days and their UV–vis spectra analyzed.

### Estimation of decay rate constants and UV–vis spectra of oxidation products

We next sought to extract pure UV–vis spectra of intermediate species O_1_, O_2_, and O_3_ using the MCR-ALS suite of applications^43^. Isolating these pure spectra from the spectra in Fig. 6 is a challenge because the latter originate from a system with continuously varying proportions of four possible components: R_1_, O_1_, O_2_, and O_3_. Chemometric techniques such as MCR-ALS can overcome this challenge, because they use matrix factorization methods combined with suitable physical and chemical constraints to deconvolute the component spectra and determine the concentrations and pure spectral profiles of each component. MCR-ALS analysis was ran on the spectral data set within a truncated wavelength range of 230–500 nm. Each spectrum was represented as a row of a matrix, and initial estimates for the concentration matrix were made using the purest variable detection method (SIMPLISMA)^43,47,48^. The system was analyzed as a four-component system comprising R_1_, O_1_, O_2_ and O_3_, with the constraints used to reduce the ambiguity in matrix decomposition being non-negativity of spectra and concentrations using a non-linear least squares method, as well as a stoichiometric balance closure constraint. A hard constraint for following a first-order kinetic model was also used. As a control, we ran MCR-ALS analysis on *operando* UV–vis spectra taken on a ferrocyanide-based electrolyte during bulk electrolytic cycling (Supplementary Fig. 9), and demonstrated that the technique successfully infers the correct spectral profiles and concentrations for ferrocyanide and ferricyanide (Supplementary Fig. 10) (Supplementary Section 3 for more details).

**Fig. 7:**
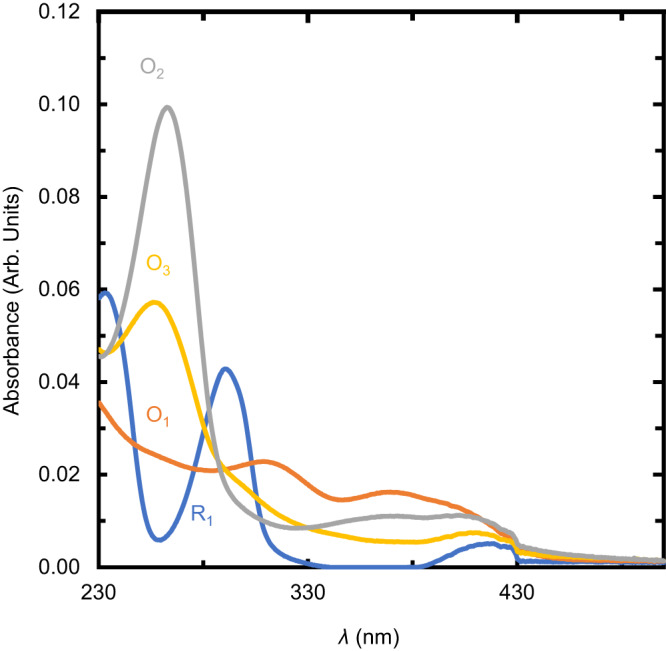
Inference of UV-vis spectra using MCR-ALS. Estimated spectra of R_1_, O_1_, O_2_, and O_3_ from MCR-ALS with [BQDS]_0_ = 8 mM.

Estimates for the rate constant for electrochemical conversion of R_1_ to O_1_ (*k*_0_), *k*_1_, and *k*_2_ are shown in Table 2. Figure 7 shows the estimated spectra from the experiment with [BQDS]_0_ = 8 mM. The estimated spectral profiles indicate that O_2_ and O_3_ have similar spectra, with O_3_ having a weaker peak centered around 260 nm compared to O_2_. This finding makes sense of the observation from the experimental UV–vis spectra that the peak at 260 nm rose and then fell during oxidative electrolysis of the BQDS electrolyte (Supplementary Fig. 8e). The larger variance for *k*_2_ than *k*_1_ is likely due to this phenomenon as well; the overlap in peak position for O_2_ and O_3_ limits the ability of MCR-ALS to distinguish between the two species. On the other hand, the rate constants *k*_0_ and *k*_1_ are relatively well determined and correspond to time constants on the order of a minute and half an hour, respectively. The estimated concentration profiles for R_1_, O_1_, O_2_, and O_3_ for an [BQDS]_0_ = 8 mM and other concentrations are shown in Supplementary Fig. 11 and the estimated spectra are shown in Supplementary Fig. 12.

**Table 2:** Rate constants for the oxidation of BQDS and subsequent Michael attack determined using MCR-ALS performed on the in-line UV–vis data from *operando* flow cells

[BQDS]_0_	*k*_0_(10^−2^*s*^−1^)	${\sigma }_{{k}_{0}}(1{0}^{-5}{s}^{-1})$	*k*_1_(10^−4^*s*^−1^)	${\sigma }_{{k}_{1}}(1{0}^{-7}{s}^{-1})$	*k*_2_(10^−5^*s*^−1^)	${\sigma }_{{k}_{2}}(1{0}^{-9}{s}^{-1})$
5 mM	3.76	9.19	3.92	9.06	2.57	37.6
8 mM	4.68	5.67	5.67	7.11	0.78	7.56
15 mM	1.24	6.76	4.78	6.76	2.25	19.7

The mean values for *k*_1_ and *k*_2_ are plotted in Fig. 4 and superimposed on the uncertainty-quantified estimates of *k*_1_ and *k*_2_ obtained via Bayesian inference and those from MCR-ALS analysis on the ex situ BQDS decay data. Within uncertainty, there is good agreement among both sets of estimates, leading to the conclusion that reactant decay and thus capacity fade within a flow cell containing BQDS in the CLE is largely driven by chemical decay of the BQDS electrolyte based on the mechanism in Fig. 1.

### Discussion

The decomposition mechanism in Fig. 1, which was first proposed in a study by Yang et al.^40^, was originally formulated based on NMR measurements of aliquots from a BQDS electrolyte after 2, 120 and 400 charge-discharge cycles in a flow cell. NMR is a more chemically specific technique than UV–vis, but it is difficult to quantify decay kinetics from such NMR measurements because if capacity fade is driven by a chemical decay—as has now been determined—the amount of time oxidized BQDS (or successive oxidized derivatives) are present in solution is a more relevant metric rather than the number of cycles spent in a flow cell^8^. Therefore, to verify that the water addition steps in Fig. 1 proceeded under potentiostatic conditions, we set up a flow cell with a BQDS-containing CLE and Methylene Blue counter electrolyte, and, over the course of 24 h, drew aliquots from the CLE while it was under a fixed oxidative potential of 0.8 V.

The results of this experiment are shown in Supplementary Fig. 13a. The spectrum for BQDS contains two singlets at 7.17 and 7.41 ppm, corresponding to protons at the two unsubstituted aryl positions^40,44^. After 1 hour, a singlet at 7.06 ppm is dominant, corresponding to the proton at the only unsubstituted aryl position in O_2_. After 24 h, the peak corresponding to the aromatic proton in O_2_ disappeared, and no aromatic proton peaks were visible in the NMR scan, which would be expected given that all aryl positions are substituted in O_3_. The evolution in NMR spectra and relative shifts in peak position are in line with our expectations based on NMR spectra simulated for O_1_, O_2_ and O_3_ using ChemDraw (Supplementary Fig. 13b).

**Fig. 8:**
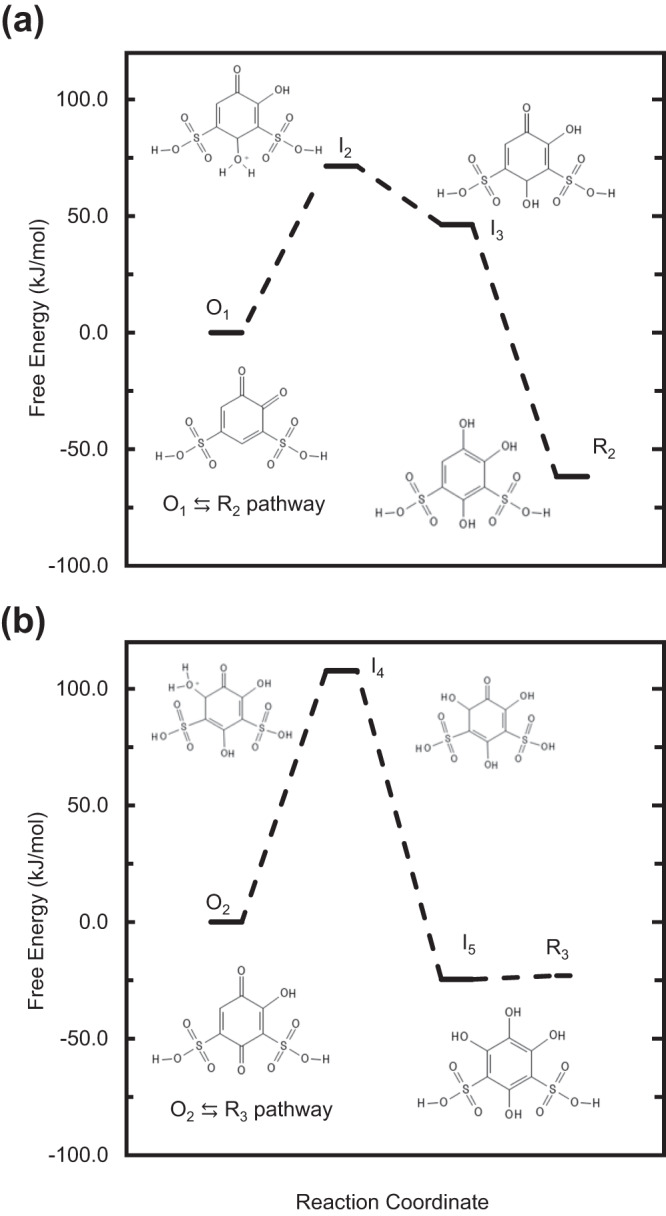
DFT calculations of energetic stability. DFT-computed reaction free energy diagram for decomposition of (**a**) O_1_ to R_2_ and (**b**) O_2_ to R_3_ at standard conditions.

We also predicted the decomposition reaction thermodynamics for conversions of O_1_ to R_2_ and O_2_ to R_3_ using DFT modeling with the B3LYP functional and COSMO implicit solvation (Fig. 8). The Brønsted–Evans–Polanyi (BEP) principle observes that the difference in activation energy between two reactions of the same family is proportional to the difference of their reaction energies. Assuming the principle holds, DFT calculations of the reaction energies allow the kinetics of the two reactions responsible for capacity fade, O_1_ to R_2_ and O_2_ to R_3_, to be compared qualitatively. The O_1_ to R_2_ reaction (Fig. 8a) is more thermodynamically favored than O_2_ to R_3_ (Fig. 8b), with overall free energy changes of −61.7 kJ/mol versus −23.0 kJ/mol, respectively. The first elementary step of the O_2_ to R_3_ pathway is much more endothermic (ΔG_O2→I4_ = 107.8 kJ/mol) than the first elementary step of the O_1_ to R_2_ pathway (i.e., ΔG_O1→I2_ = 71.4 kJ/mol). Subsequent steps for both reactions are downhill in free energy or essentially thermoneutral (i.e., I_5_ to R_3_). Thus, the first elementary step of O_2_ to I_4_ likely has the highest activation energy for both pathways based on the BEP principle. It follows that the O_1_ to R_2_ decomposition should occur more rapidly than that of O_2_ to R_3_ (*k*_2_ < *k*_1_), which is consistent with the results of applying MCR-ALS and Bayesian inference to our spectroscopic data. The DFT-predicted energetics qualitatively follow what is expected for apparent activation barriers calculated from the experimental values for *k*_1_ and *k*_1_ obtained in Sections “Model selection and uncertainty-quantified rates of Michael attack using Bayesian inference and multivariate curve resolution analysis” and “Estimation of decay rate constants and UV–vis spectra of oxidation products”. Assuming transition state theory, and a pre-exponential factor of *k*_*b*_*T*/*h* = 6 × 10^12^ s^−1^, we obtained activation barriers of 86 and 91 kJ/mol for the conversion of O_1_ to R_2_ and O_2_ to R_3_, respectively.

Taken together, our DFT computations and NMR results strongly corroborate conclusions from Sections “Model selection and uncertainty-quantified rates of Michael attack using Bayesian inference and multivariate curve resolution analysis” and “Estimation of decay rate constants and UV–vis spectra of oxidation products”. Thus, combining UV–vis spectroscopy—whether ex situ or from *operando* flow cells—with matrix-based curve resolution, as well as Bayesian model selection and parameter inference, is a viable strategy for elucidating the role of Michael attack in the decomposition of flow battery reactants. Importantly, our techniques are able to resolve processes occurring across a range of timescales, from a minute to several hours. In the context of ongoing efforts toward designing Michael attack-resistant quinone-based posolytes^40,49,50^, the methods demonstrated in this paper might be extended in future work toward high-throughput screening of other high-potential quinone derivatives^45^, or evaluating the correlation between the rate of Michael attack and a quinone’s redox potential^51^. Moreover, UV–vis signatures of intermediate species inferred from *operando* spectroscopy may be compared against ex situ spectra of decay species that have been proposed to feature in one or more hypothesized decay mechanisms. These campaigns may be extended to other molecular classes that are subject to Michael attack, and which are currently under consideration for organic flow battery applications, such as quinoxalines^52^ and biphenols^53^.

More generally, the combination of spectrophotometry with statistical inference techniques as depicted by the workflow in Fig. 1 can be deployed in connection with a wider variety of decomposition mechanisms to which flow battery reactants from other molecular classes are susceptible. Although it might be difficult to use these methods to determine a completely unknown degradation mechanism, they may be used to verify a proposed hypothesis about the link between molecular decay and capacity fade, choose between competing hypotheses (which might be found based on techniques such NMR spectroscopy and mass spectrometry), or guide the design of cycling experiments in such a way as to extract decay rate parameters with high certainty and low experimental effort (i.e., via optimal experimental design^54^). In addition to UV–vis spectroscopy, it is also possible to apply Bayesian techniques to other forms of spectroscopic data where the signal may be proportional to concentration such as Fourier Transform Infrared (FTIR) spectroscopy. The decay rate parameters thus obtained may in turn be supplied to analytical or numerical electrochemical models that can simulate capacity retention for a given load/duty cycle^55,56^, thus paving the way to flow battery lifetime predictions. Even though we conduct our analysis for concentrations below 20 mM, we determined reaction kinetic rates and orders, so it should be possible to apply these to predict decay rates when practical concentrations (>0.5 M) are used. Beyond capacity fade in organic RFBs, these methods may also be deployed for other flow cell-based applications in which charge transfer is accompanied by homogeneous chemical conversion(s), such as electrosynthesis^57^ or (electro)chemical separations (e.g., CO_2_ capture)^58^.

## Methods

### Chemicals

BQDS, H_2_SO_4_, potassium dichromate, D_2_O, CH_3_SO_3_D and Methylene Blue (MB) were procured from Sigma Aldrich and used as received.

### Spectroscopic measurement of chemical oxidation of BQDS and Michael attack

UV–vis measurements of Michael attack of BQDS were conducted by monitoring the optical absorbance of K_2_Cr_2_O_7_ after the addition of a known quantity of BQDS. For each measurement, an aqueous solution of 2 mL of K_2_Cr_2_O_7_ was first prepared in a polystyrene cuvette with a 10 mm path length and 1 M H_2_SO_4_ as supporting electrolyte; then BQDS was added to the solution and UV–vis spectra were recorded every 5 min. All UV–vis spectra were recorded at room temperature using a DH-mini light source from Ocean Insight and an HDX detector.

### Flow cell preparation

Flow cells were constructed with cell hardware from Fuel Cell Technologies (Albuquerque, New Mexico), and assembled into a zero-gap configuration, similar to a previous report^59^. Pyrosealed POCO graphite flow plates with interdigitated flow patterns were used for both electrodes. Each electrode comprised a 5 cm^2^ stack of one sheet of CE Tech GF020 graphite felt (Fuel Cell Store, uncompressed thickness of 2.0 mm). All electrodes were baked in air for 12 h at 400 °C prior to use. The outer portion of the space between the electrodes was gasketed by Viton sheets with the area over the electrodes cut out. A 120-μm-thick Nafion 115 (Fuel Cell Store) ion-exchange membrane was used as the separator in the BQDS-MB flow cells with and without in-line UV–vis monitoring, and torque applied during cell assembly was 13.6 Nm on each of the eight bolts. A Cole-Parmer Masterflex peristaltic pump circulated the electrolyte through the flow cell through fluorinated ethylene propylene tubing of 1.61 mm inner diameter. We obtained calibration curves for each pump that enabled translation from revolutions per minute to a volumetric flow rate in mL/min. For the flowcell experiments, we used a volumetric flow rate of 30 mL/min. A Biologic VSP-300 potentiostat was used for all cell cycling, voltammetry, and impedance spectroscopy measurements.

### In-line *operando* UV–vis measurements

We monitored the rate of Michael attack of BQDS during its oxidation in an *operando* flow cell. This measurement was done by setting up an aqueous flow cell with a capacity-limiting electrolyte (CLE) comprising 8 mL of between 5 and 15 mM of BQDS, and directing the flow of electrolyte through an absorbance micro cross flow cell (Supplementary Fig. 14) from Firebird Optics. In all cases, 20 mL of 20 mM of Methylene Blue (MB) was used as the redox-active material in the non-capacity-limiting (NCLE) (or counter) electrolyte. We used MB in the counter electrolyte because it has been shown to have higher chemical stability as measured from symmetric flow cell cycling^60^ than anthraquinone 2,7-disulfonic acid^61^, which has been previously deployed in the negolyte of BQDS full cells. The lower voltage of the BQDS-MB flow cell (0.15 V) compared to a BQDS-AQDS cell (~0.65 V) did not hinder our objective of investigating the degradation of BQDS. Our goal was to have a counter electrolyte with adequate excess capacity and stable redox behavior, not a high potential full-cell. The supporting electrolyte comprised 0.9 M H_2_SO_4_ and 0.1 M CH_3_COOH; the CH_3_COOH was used to prevent MB from coagulating^62^.

Although practical flow batteries may require the use of reactants with concentrations in excess of 0.5 M, we are constrained to lower concentrations due to the saturation limit of the spectrometer. Given that BQDS has a molar absorptivity of 2.4 × 10^−3^ M^−1^ cm^−1^ (considering the peak at 234 nm), we would need an impractically short path length of 1.2 × 10^−3^ cm or lower due to the saturation limit of the spectrometer if a 0.5 M concentration is to be used. This limitation does not exist for the in situ technique, where aliquots can be periodically drawn from the CLE and diluted before UV–vis analysis. In situ UV–vis measurements may therefore readily permit analysis of reactant concentrations >0.5 M, but at the expense of a lower time resolution than more continuous, *operando* measurements.

For each measurement, unless specified otherwise, UV–vis spectra were recorded every 5 s for the first hour, and then every 30 s for the next 10–20 h. The spectrometer and detector used for this experiment were the same as the ones used in Section “Spectroscopic measurement of chemical oxidation of BQDS and Michael attack”. BQDS was oxidized potentiostatically at a cell potential of 0.8 V, which is 0.6 V higher than the nominal potential of a BQDS-MB flow cell.

### NMR measurements and simulations

150 μL aliquots of a BQDS electrolyte under oxidation in a BQDS-MB flow cell were taken intermittently and examined using Nuclear Magnetic Resonance (NMR) spectroscopy. The CLE comprised 100 mM of BQDS, whereas the counter electrolyte comprised 50 mM of Methylene Blue (MB) with a six-fold excess capacity. The cell was charged at a constant current of 0.5 A until a voltage of 1 V was obtained, after which it was held at 1 V for 24 h. Aliquots were retrieved from the electrolyte after 5, 10, 20, 30, 60, 300, and 1440 min from the start of oxidation. Prior to NMR analysis, each aliquot was diluted to 20 mM with deuterated water (D_2_O) as the solvent and 50 mM CH_3_SO_3_D was added as an internal standard.

NMR measurements were conducted on a Varian MR400 400 MHz (9.4 Tesla) Premium Shielded Magnet NMR spectrometer. The H-NMR simulations for BQDS derivatives were obtained using CDCl_3_ as the solvent under a frequency of 300 MHz with the ChemDraw software version 18.2.

### Bayesian inference and model selection

In this section we present a general Bayesian framework for parameter inference and model selection. We then use these methods for our models and experiments in Section “Results and discussion”.

#### Bayesian parameter inference

Bayesian parameter inference is exercised on a given model ${{{{{{{\mathcal{M}}}}}}}}$, such as the BQDS decay model in Fig. 1. We denote the collection of all model parameters (e.g., reaction orders and rate constants) by *θ*, and all measurements (e.g., absorbance data obtained using UV–vis spectroscopy) by *y*. Under a Bayesian framework, the uncertainty of *θ* is represented by a probability density function (PDF). When new measurements are acquired, the uncertainty of *θ* is updated through Bayes’ rule:

7 $$p(\theta|y,\,{{{{{{{\mathcal{M}}}}}}}})=\frac{p(y|\theta,\,{{{{{{{\mathcal{M}}}}}}}})p(\theta|{{{{{{{\mathcal{M}}}}}}}})}{p(y|{{{{{{{\mathcal{M}}}}}}}})},$$

where $p(\theta|{{{{{{{\mathcal{M}}}}}}}})$ is the prior PDF representing the initial uncertainty of *θ* in model ${{{{{{{\mathcal{M}}}}}}}}$ before having the new measurements *y*, and $p(\theta|y,\,{{{{{{{\mathcal{M}}}}}}}})$ is the posterior PDF representing the updated uncertainty after incorporating *y*. Furthermore, $p(y|\theta,\,{{{{{{{\mathcal{M}}}}}}}})$ is the likelihood PDF that provides a probabilistic description of the discrepancy between the model’s predictions and experimental observations, and $p(y|{{{{{{{\mathcal{M}}}}}}}})=\int\,p(y|\theta,\,{{{{{{{\mathcal{M}}}}}}}})p(\theta|{{{{{{{\mathcal{M}}}}}}}})\,d\theta$ is the Bayesian model evidence (also called the marginal likelihood) that acts as a normalization constant to ensure $p(\theta|y,{{{{{{{\mathcal{M}}}}}}}})$ remains a proper PDF (integrates to unity). Solving the Bayesian inference problem then entails computing or characterizing the posterior $p(\theta|y,{{{{{{{\mathcal{M}}}}}}}})$.

The prior $p(\theta|{{{{{{{\mathcal{M}}}}}}}})$ may be chosen in either a non-informative or informative manner. In the former, for example, if reasonable lower and upper bounds are known for the model parameter, then a uniform distribution can be imposed across this range. Such “flat” distribution means that initially all values are equally probable and one does not favor any particular region; this non-informative prior also appeals to the Principle of Maximum Entropy^63^. In the latter, if historical data or domain experts are available, the prior PDF can be formed to incorporate this knowledge through techniques of expert elicitation^28^. In our work, we take a non-informative approach and adopt a uniform prior distribution.

The likelihood $p(y|\theta,{{{{{{{\mathcal{M}}}}}}}})$ quantifies how likely one is to observe *y* if the model’s parameter values were actually *θ*. Effectively, it provides a probability measure on the discrepancy between a model’s predictions and experimental observations, which may be induced by measurement noise or modeling error. We employ a commonly used likelihood form that involves additive Gaussian error:

8 $$y={g}_{{{{{{{{\mathcal{M}}}}}}}}}(\theta )+\epsilon,$$

where ${g}_{{{{{{{{\mathcal{M}}}}}}}}}(\theta )$ is model ${{{{{{{\mathcal{M}}}}}}}}$’s prediction on the observable quantities if parameter values were *θ*, and *ϵ* is a zero-mean Gaussian distribution ${{{{{{{\mathcal{N}}}}}}}}(0,\,{{{\Sigma }}}_{\epsilon })$. Subsequently, the likelihood PDF may be evaluated as $p(y|\theta,\,{{{{{{{\mathcal{M}}}}}}}})={p}_{\epsilon }(y-{g}_{{{{{{{{\mathcal{M}}}}}}}}}(\theta ))={{{{{{{\mathcal{N}}}}}}}}(y-{g}_{{{{{{{{\mathcal{M}}}}}}}}}(\theta );0,{{{\Sigma }}}_{\epsilon })$. Whereas such a zero-mean error assumption is commonly justified for measurement error from instruments, it is often inadequate to incorporate modeling error (i.e., systematic error due to incorrect assumptions in a model, such as the wrong number of Michael addition events to oxidized BQDS); however, this issue is separately addressed through Bayesian model selection in the next section.

Lastly, the Bayesian inference problem requires solving for the posterior. The posterior PDF is generally intractable to compute. Instead, one can characterize the posterior by generating samples from it, most commonly via Markov chain Monte Carlo (MCMC) methods^64,65^. We therefore performed posterior sampling using the Metropolis–Hastings algorithm^66,67^ from PyMC3, a mature probabilistic programming Python package that can handle high dimensional parameter settings^68^.

#### Bayesian model selection

Bayesian parameter inference from the previous section provides a means to quantify parameter uncertainty for a given model ${{{{{{{\mathcal{M}}}}}}}}$. However, what if we wish to compare different models that are built on different assumptions, such as BQDS decay models with one versus two versus three Michael additions? Such a task of model selection can also be accomplished in a Bayesian framework^29,30^. Among *n*_*M*_ candidate models, the probability that the *i*th model ${{{{{{{{\mathcal{M}}}}}}}}}_{i},i=1,\ldots,{n}_{M}$ is the true data-generating model of experimental observations *y* can also be expressed through Bayes’ rule:

9 $${\mathbb{P}}({{{{{{{{\mathcal{M}}}}}}}}}_{i}|y)=\frac{p(y|{{{{{{{{\mathcal{M}}}}}}}}}_{i}){\mathbb{P}}({{{{{{{{\mathcal{M}}}}}}}}}_{i})}{p(y)},$$

where ${\mathbb{P}}({{{{{{{{\mathcal{M}}}}}}}}}_{i})$ is the prior probability mass function (PMF) of model ${{{{{{{{\mathcal{M}}}}}}}}}_{i}$, and ${\mathbb{P}}({{{{{{{{\mathcal{M}}}}}}}}}_{i}|y)$ is posterior PMF given observations *y*. The new model-likelihood term $p(y|{{{{{{{{\mathcal{M}}}}}}}}}_{i})$ is in fact the model evidence (denominator term) from Eq. (7). $p(y)=\mathop{\sum }\nolimits_{i=1}^{{n}_{M}}p(y|{{{{{{{{\mathcal{M}}}}}}}}}_{i}){\mathbb{P}}({{{{{{{{\mathcal{M}}}}}}}}}_{i})$ still serves as a normalization constant but can be computed easily because ${{{{{{{{\mathcal{M}}}}}}}}}_{i}$ is discrete. If a uniform model prior is used—i.e., all models are initially equally probable with ${\mathbb{P}}({{{{{{{{\mathcal{M}}}}}}}}}_{i})=1/{n}_{M}$—it follows that ${\mathbb{P}}({{{{{{{{\mathcal{M}}}}}}}}}_{i}|y)=p(y|{{{{{{{{\mathcal{M}}}}}}}}}_{i}) / \mathop{\sum }\nolimits_{j=1}^{{n}_{M}}p(y|{{{{{{{{\mathcal{M}}}}}}}}}_{j})$. The key to Bayesian model selection is therefore estimating the model evidence term $p(y|{{{{{{{{\mathcal{M}}}}}}}}}_{i})$.

We elect to use a direct Monte Carlo integration to estimate the model evidence:

10 $$P(y|{{{{{{{{\mathcal{M}}}}}}}}}_{i})=\int\,p(y|{\theta }_{i},\,{{{{{{{{\mathcal{M}}}}}}}}}_{i})p({\theta }_{i}|{{{{{{{{\mathcal{M}}}}}}}}}_{i})\,d{\theta }_{i}\approx \frac{1}{N}\mathop{\sum }\limits_{j=1}^{N}p(y|{\theta }_{i}^{(j)},\,{M}_{i})$$

where *θ*_*i*_ denotes parameters of model ${{{{{{{{\mathcal{M}}}}}}}}}_{i}$, and ${\theta }_{i}^{(j)},j=1,\ldots,N$ represents the *j*th sample of *θ*_*i*_ drawn from the parameter-prior distribution $p({\theta }_{i}|{{{{{{{{\mathcal{M}}}}}}}}}_{i})$. This Monte Carlo estimator for the model evidence may endure high variance if the posterior concentrates narrowly within the prior (i.e., many of the prior samples may have a near-zero contribution to the summand in Eq. (10)). More advanced techniques can be employed to improve the estimate, such as using importance sampling (e.g., Ch. 3.3 of ref. ^69^) or variational inference^70^; we leave the exploration of these advanced numerical methods for future work. The Bayesian methodology also has limitations. For example, the MCMC sampling algorithm for performing Bayesian inference can take a long time to mix (converge) if the posterior is sharply multimodal; in our work we mitigate this effect using multiple chains with randomized starting points. Computing the probabilities of several hypotheses using Bayesian model selection can also require many samples to stabilize and can be computationally expensive. Furthermore, Bayesian model selection is a method that identifies the most probable hypothesis from a given candidate set; it is not designed to generate new hypotheses.

### Multivariate curve resolution analysis

We determined the UV–vis spectra of all intermediate species that evolved during BQDS oxidation, and calculated the rate constants of Michael attack of oxidized BQDS using the MCR alternating least squares (MCR-ALS) suite of applications^43^. The MCR-ALS technique is based on the matrix form of the Beer–Lambert Law:

11 $$X=C{S}^{T}$$

where *X* is a matrix containing the spectra taken at each time step, *C* is the matrix whose columns contain the concentration of each independent component in the system with time, and *S* is the matrix that contains the pure spectra (the product of molar absorption coefficient and path length) of each component in the system along its rows. The dimensions of *X* are *t* × *λ*, where *λ* is the number of wavelengths recorded by the UV–vis spectrometer, and *t* is the number of spectra recorded; the dimensions of *C* are *t* × *n*, where *n* is the number of components in the system; and the dimensions of *S*^*T*^ are *n* × *λ*. The Beer’s Law decomposition of a spectral matrix is illustrated in Supplementary Fig. 15.

MCR-ALS uses the alternating least squares technique for matrix decomposition to determine the matrices *C* and *S*, thus allowing determination of the pure spectra for each of the components in the system and the associated rate constants of decomposition. Initial guesses for the factored matrices can be obtained using either evolving factor analysis (EFA) or purest variables detection (SIMPLISMA) techniques, followed by the iterative alternating least squares procedure to find the best fitting matrices^47,48,71^. In this study we used the SIMPLISMA method. We also imposed additional constraints of non-negativity in spectra and concentrations, and closure (i.e., a fixed total concentration for all species) in order to reduce ambiguities in the matrix factorization. A key limitation of MCR-ALS is that it is only valid (assumes) for first order reactions; however this assumption is verified by our Bayesian model selection results.

### Density functional theory calculations

All DFT calculations were performed in the NWChem computational chemistry software version 7.0.0^72^. Gibbs free energies of the species in the BQDS decomposition pathway were predicted. Each species in the pathway was geometry optimized using the B3LYP functional. The 6-311G** basis set was used^73,74^. The COSMO model with default parameters was used to implicitly treat molecule solvation by water^75^. The convergence criteria in NWChem used were ${{{{{{{\rm{GMAX}}}}}}}}=0.00045$, GRMS = 0.00030, ${{{{{{{\rm{XMAX}}}}}}}}=0.00180$, XRMS = 0.00120, and the electronic energy convergence was 10^−5^ hartree. Vibrational, rotational, and translational enthalpic and entropic contributions (assuming an ideal gas with rigid rotor-harmonic oscillator) were included to estimate the free energies at standard state.

## Supplementary information

Supplementary Information

## Data Availability

The code and data for Bayesian inference analysis have been deposited on the GitHub repository via 10.5281/zenodo.7807364, and the DFT files and data are available in the NOMAD repository via 10.17172/NOMAD/2023.01.14-1.
