# Supplementary material for: Understanding capacity fade in organic redox-flow batteries by combining spectroscopy with statistical inference techniques
Source: Nat Commun. 2023 Jun 16;14:3602. doi: 10.1038/s41467-023-39257-z (PMC10275907; doi:10.1038/s41467-023-39257-z)
Supplement: Supplementary file 1 — Supplementary Information [file 41467_2023_39257_MOESM1_ESM.pdf]

Supplementary Information

Inventory of Supplementary information

Supplementary Figure 1 related to Figure 2a, b and c.
Supplementary Figure 2 related to Figure 2a, b and c.
Supplementary Figure 3 related to Figure 2a, b and c.
Supplementary Figure 4 related to Figure 2a, b and c.
Supplementary Figure 5 related to Figure 2a, b and c.
Supplementary Figure 6 related to Figure 5.
Supplementary Figure 7 related to Figure 5.
Supplementary Figure 8 related to Figure 7.
Supplementary Figure 9 related to Figure 7
Supplementary Figure 10 related to Figure 7
Supplementary Figure 11 related to Figure 6
Supplementary Figure 12 related to Figure 6
Supplementary Figure 13 related to the NMR characterization
Supplementary Figure 14 related to *operando* UV-vis characterization
Supplementary Figure 15 related to MCR-ALS analysis
Supplementary Equations 1,2 and 3 are related to Eqns. 3, 4, and 5.

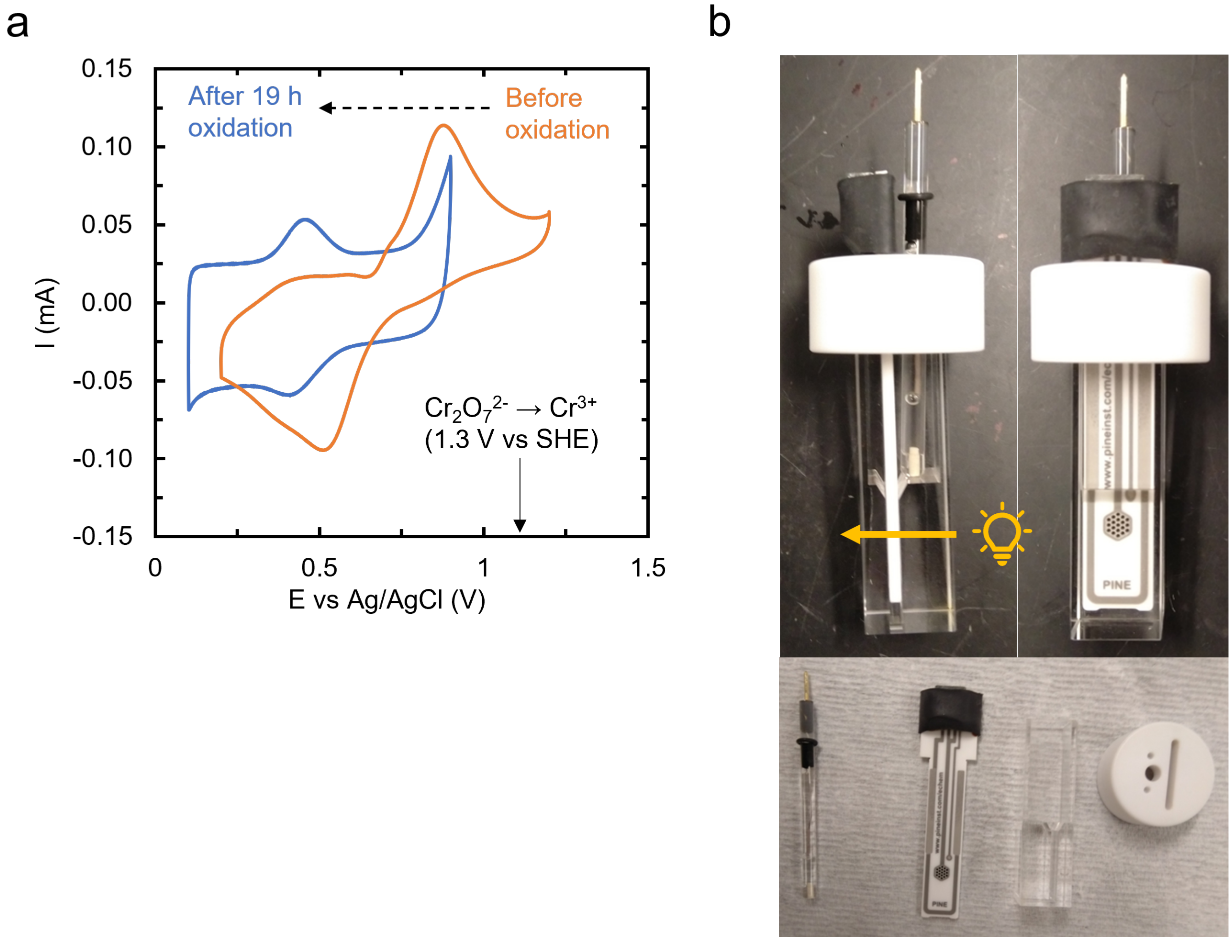

Supplementary Figure 1. (a) Cyclic voltammograms of a freshly prepared solution of 0.3 mM BQDS and after oxidation for 19 hours at 0.95 V vs Ag/AgCl in a spectroelectrochemical cell. (b) A photograph of the spectroelectrochemical cell. The spectroelectrochemical cell has a printed platinum honeycomb mesh (allowing light to pass through it) as a working electrode with a counter electrode integrated into it with a path length of 1.7 mm.

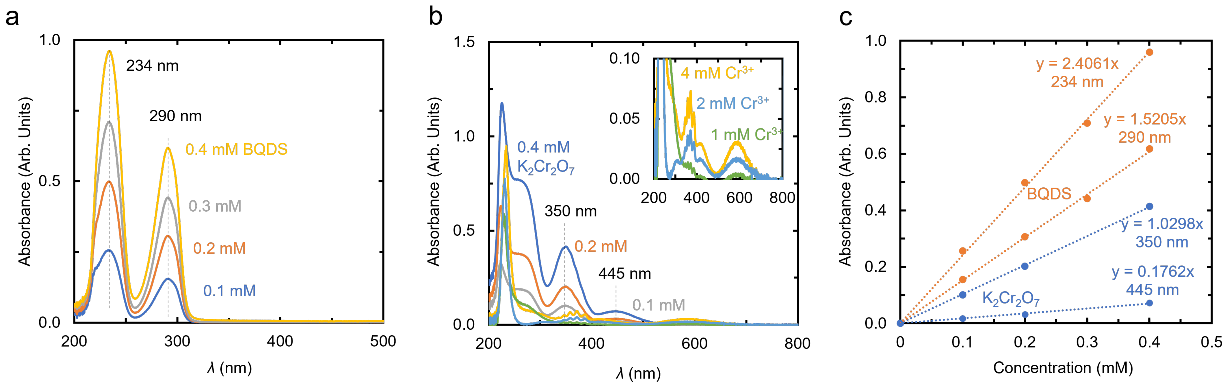

Supplementary Figure 2. UV-vis spectra of (a) BQDS and (b) $K_2Cr_2O_7$ at 0.1, 0.2, 0.3 and 0.4 mM. (c) Calibration curves for BQDS and $K_2Cr_2O_7$ using absorbance at 234 and 290 nm for BQDS and 350 and 445 nm for $K_2Cr_2O_7$.

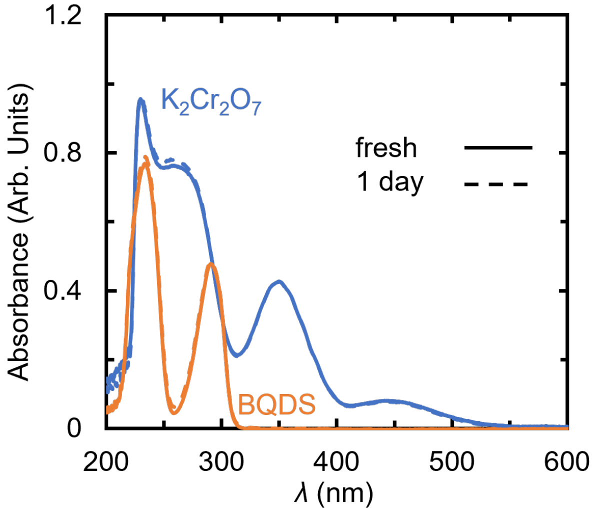

Supplementary Figure 3. UV-vis signatures of freshly prepared vs 1 day-old solutions of 0.4 mM $K_2Cr_2O_7$ and 0.3 mM BQDS in 1 M H_2SO_4.

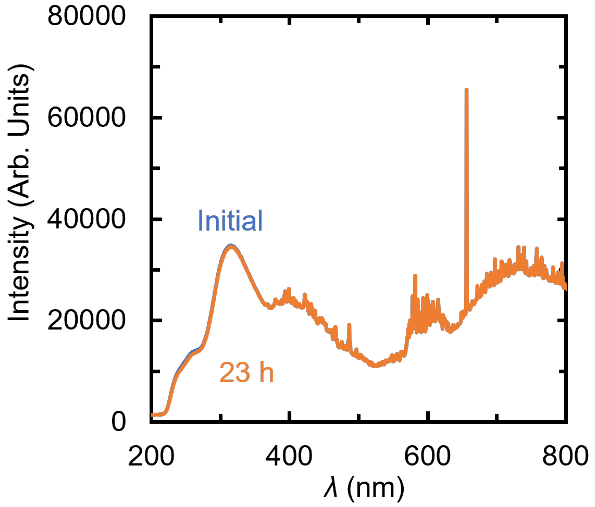

Supplementary Figure 4. UV-vis spectrum of the lamp showing no observable change after 23 hours of operation, with the initial spectrum recorded after 1 hour after the lamp was switched on to allow the lamp to warm up.

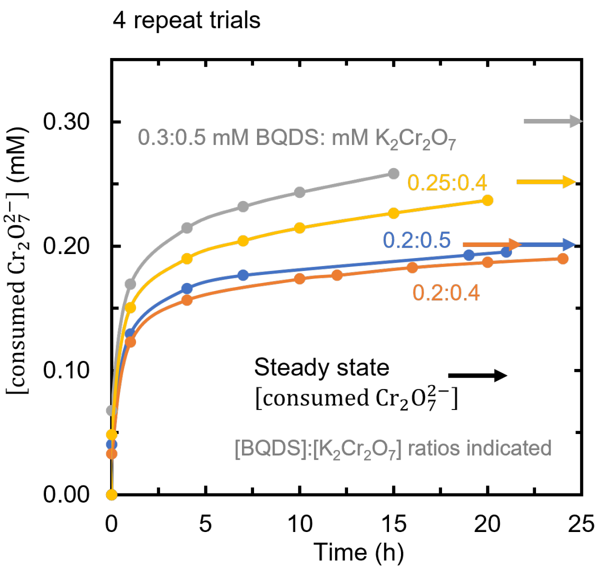

Supplementary Figure 5. Estimates for the concentration of $K_2Cr_2O_7$ consumed due to Michael attack for the four combinations of initial BQDS and $K_2Cr_2O_7$ concentrations in Fig. 2. [$K_2Cr_2O_7$] was estimated from the UV-vis peak intensity at 350 nm. The arrows depict the total amount of $K_2Cr_2O_7$ that would be expected to be consumed based on Eqn. (1).

1 First-order kinetics analytical solution

$$[\mathrm{O}_1] = [\mathrm{BQDS}]_0 e^{-k_1 t} \tag{1}$$

$$[\mathrm{O}_2] = [\mathrm{BQDS}]_0 k_1 e^{-k_1 t - k_2 t} \frac{\left(e^{k_1 t} - e^{k_2 t}\right)}{k_1 - k_2} \tag{2}$$

$$[\mathrm{O}_3] = [\mathrm{BQDS}]_0 - [\mathrm{BQDS}]_0 e^{-k_1 t} - [\mathrm{BQDS}]_0 k_1 e^{-k_1 t - k_2 t} \frac{\left(e^{k_1 t} - e^{k_2 t}\right)}{k_1 - k_2} \tag{3}$$

2 Determination of potassium dichromate concentration using MCR-ALS

A small wavelength range of 10 nm was chosen around the main potassium dichromate peak centered at 350 nm that does not have any interference from BQDS and its derivatives. The system was analyzed as a mono-component system with the initial concentration profile being determined by purest variables estimation (SIMPLISMA). Non-negativity of concentrations and spectra was enforced along with a closure constraint, namely that the concentration of potassium dichromate is always less than the corrected initial concentration used. The initial concentration had to be corrected to account for the almost-instant consumption of R_1, which does not show up in the UV-vis spectrum.

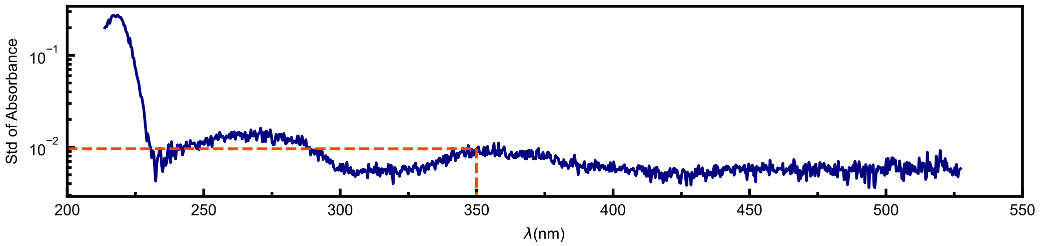

Supplementary Figure 6. Standard deviation of absorbance measurements, where the dashed red line indicates the wavelength at which we take the measurements (350 nm) and its corresponding standard deviation of absorbance (0.0096).

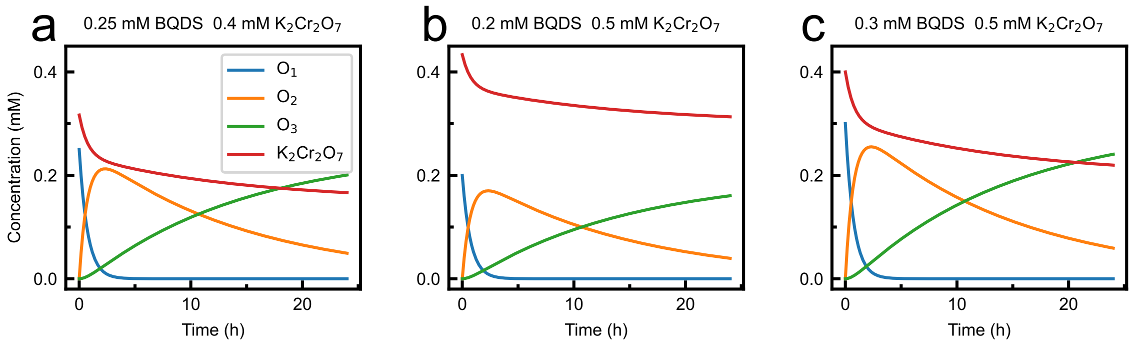

Supplementary Figure 7. Simulated evolution of oxidized species for different initial [$K_2Cr_2O_7$] and [BQDS] concentrations assuming Bayesian-inferred rate constants. (a) [BQDS] = 0.25 mM and [$K_2Cr_2O_7$] = 0.4 mM, (b) [BQDS] = 0.2 mM and [$K_2Cr_2O_7$] = 0.5 mM and (c)[BQDS] = 0.3 mM and [$K_2Cr_2O_7$] = 0.5 mM.

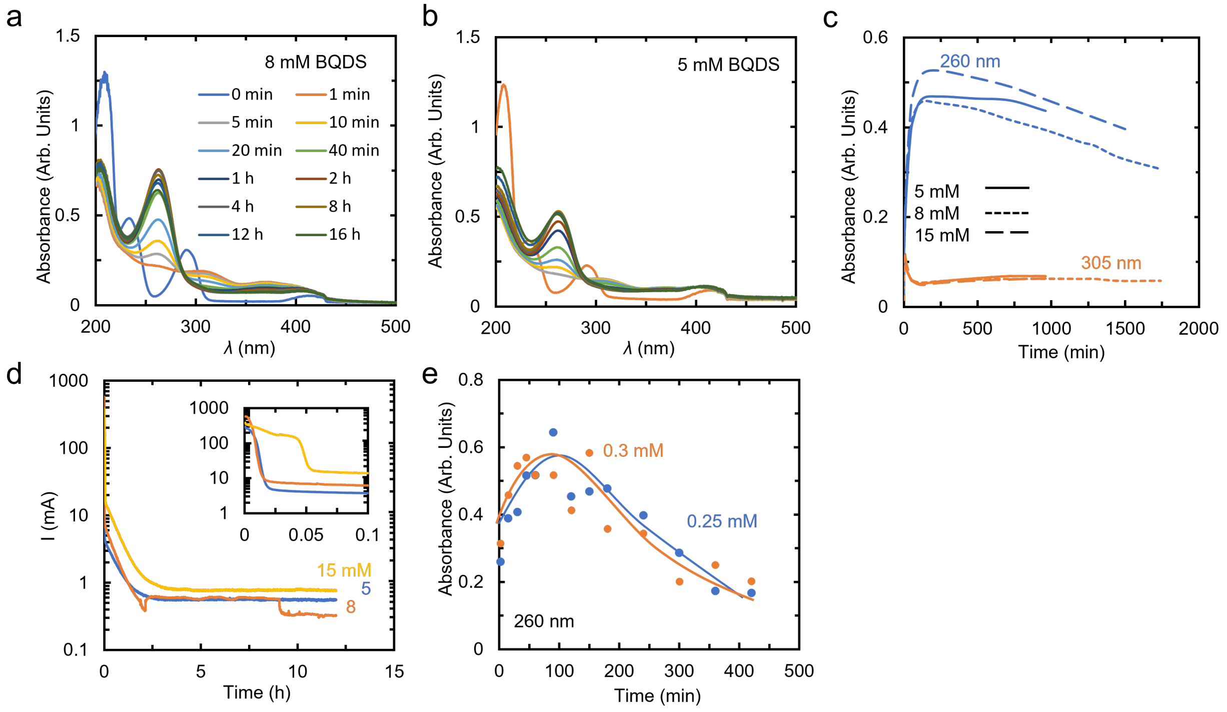

Supplementary Figure 8. Evolution of the UV-vis spectra for the CLE of the BQDS flow cell at concentrations of (a) 8 mM and (b) 5 mM. (c) Absorbance over time at 260 and 305 nm for flow cells with 15, 8, and 5 mM BQDS. (d) Evolution of current over time for all the BQDS flow cells at an oxidizing potential of 0.8 V. (e) Absorbance over time at 260 nm for *in situ* UV-vis measurements of the CLE in BQDS flow cells with BQDS concentrations of 50 and 60 mM. Aliquots for UV-vis were diluted 200× to 0.25 and 0.3 mM, respectively. Trend lines have been added to guide the eye.

3 Estimation of ferrocyanide and ferricyanide spectra using MCR-ALS

To demonstrate the robustness of the MCR-ALS spectral inference technique as applied to UV-vis spectra in an *operando* flow cell, we set up a control experiment using a flow cell with a ferrocyanide-containing CLE. The UV-vis spectra of $K_3Fe(CN)_6$ and $K_4Fe(CN)_6$ are well known, and sufficiently differentiated that they can be used to monitor the state-of-charge (SOC) of a given ferri-/ferrocyanide electrolyte. Three different cycling protocols were deployed: one in which 10 mM of both $K_3Fe(CN)_6$ and $K_4Fe(CN)_6$ were cycled in a constant-current, constant-voltage (CCCV) mode; another in which 20 mM of $K_4Fe(CN)_6$ was oxidized to $K_3Fe(CN)_6$; and a third in which $K_3Fe(CN)_6$ was reduced to $K_4Fe(CN)_6$. MCR-ALS was used with initial guesses for the spectra done using a purest variables estimation and the constraints of non-negativity of concentrations and spectra as well as closure (mass balance) of the concentration profiles. The results for the inferred spectra and the concentration profiles are presented in Supp. Fig. 10. The charge measured by the potentiostat is also shown in the CCCV data. There is an 8-second time lag between application of a current and change in the UV-vis spectrum, corresponding to the time taken by the electrolyte to flow through the manifolds of the *operando* flow cell setup. Accounting for this lag yields a very good overlap between spectroscopic (MCR-ALS derived) and coulometric (based on the amount of charge passed) SOC (inferred from $K_3Fe(CN)_6$ concentration) estimates.

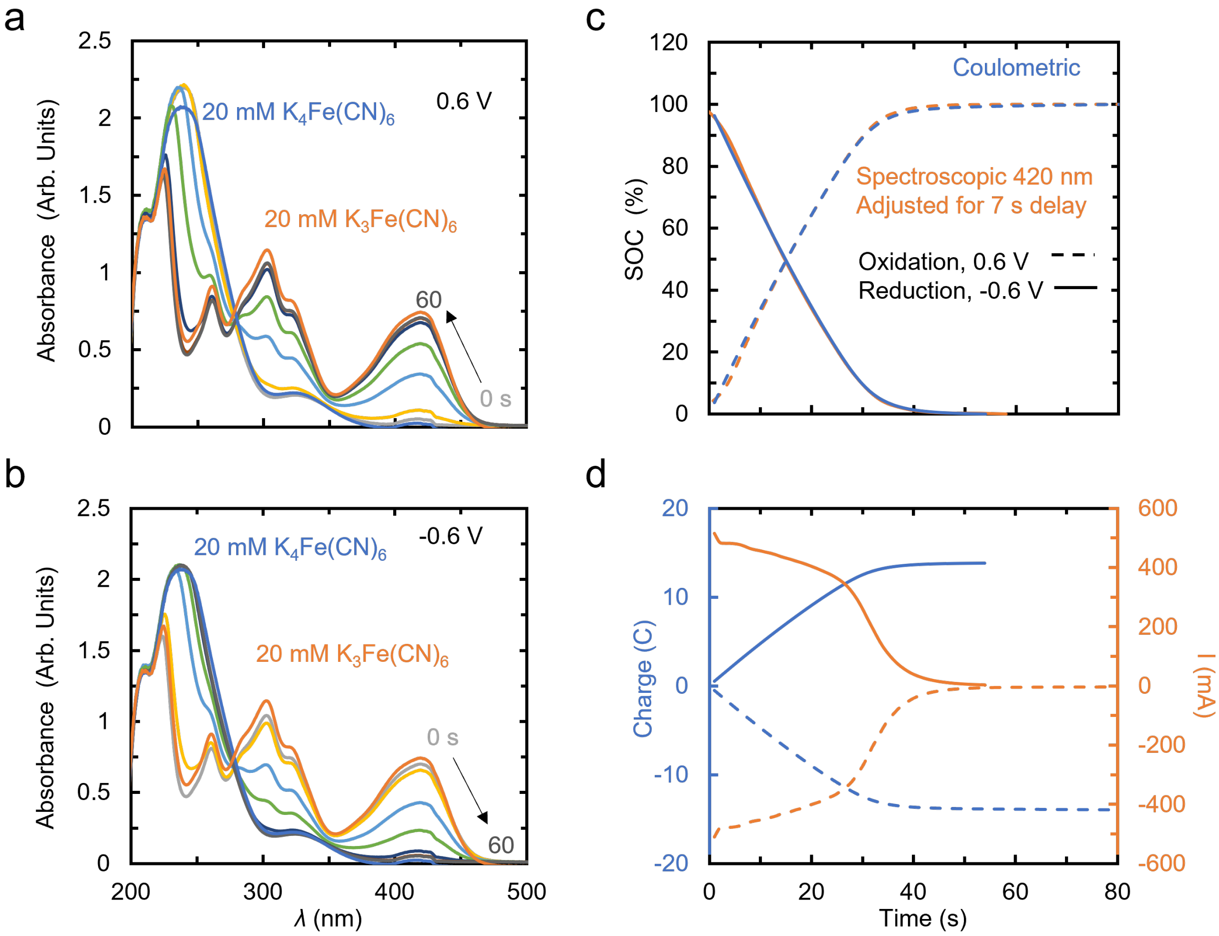

Supplementary Figure 9. UV-vis spectra from the CLE of an *operando* symmetric flow cell upon application of 0.6 V while starting from (a) 20 mM $K_4Fe(CN)_6$, and (b) 20 mM $K_3Fe(CN)_6$. (c) Estimated SOC for the experiments in (a) and (b) using Coulomb counting and spectroscopy. (d) Accumulated charge and measured current vs time for the experiments shown in (a) and (b).

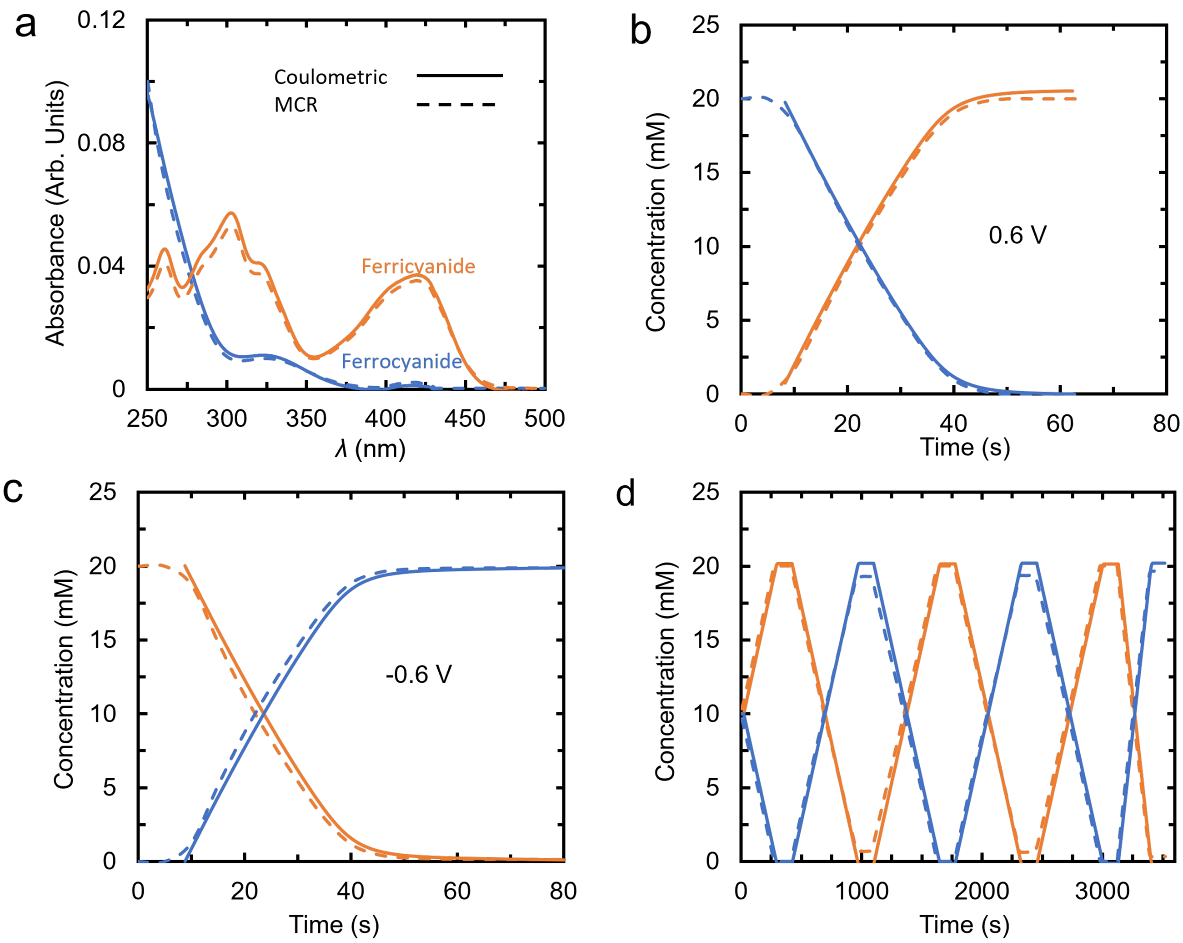

Supplementary Figure 10. (a) Predicted UV-vis spectra of ferrocyanide and ferricyanide compared to known experimental spectra. Comparison of Coulometric and MCR-ALS-derived concentration estimates for ferrocyanide and ferricyanide for applied potentials of (b) 0.6 V, resulting in $K_4Fe(CN)_6$ oxidation, (c) -0.6 V, resulting in $K_3Fe(CN)_6$ reduction, and (d) Constant current-current voltage (CCCV) cycling.

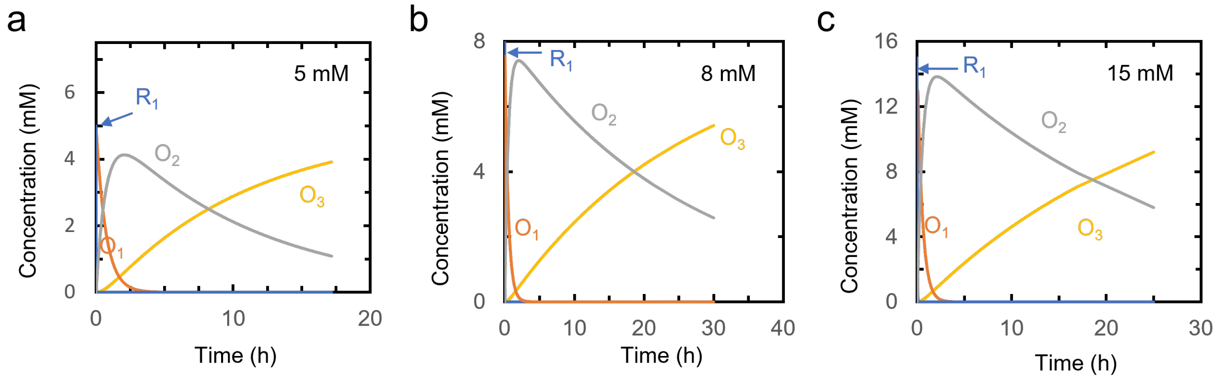

Supplementary Figure 11. Concentration profiles of R_1, O_1, O_2, and O_3 derived from MCR-ALS analysis of *operando* spectra upon potentiostatic oxidation of BQDS in a flow cell at concentrations of (a) 5 mM, (b) 8 mM, and (c) 15 mM.

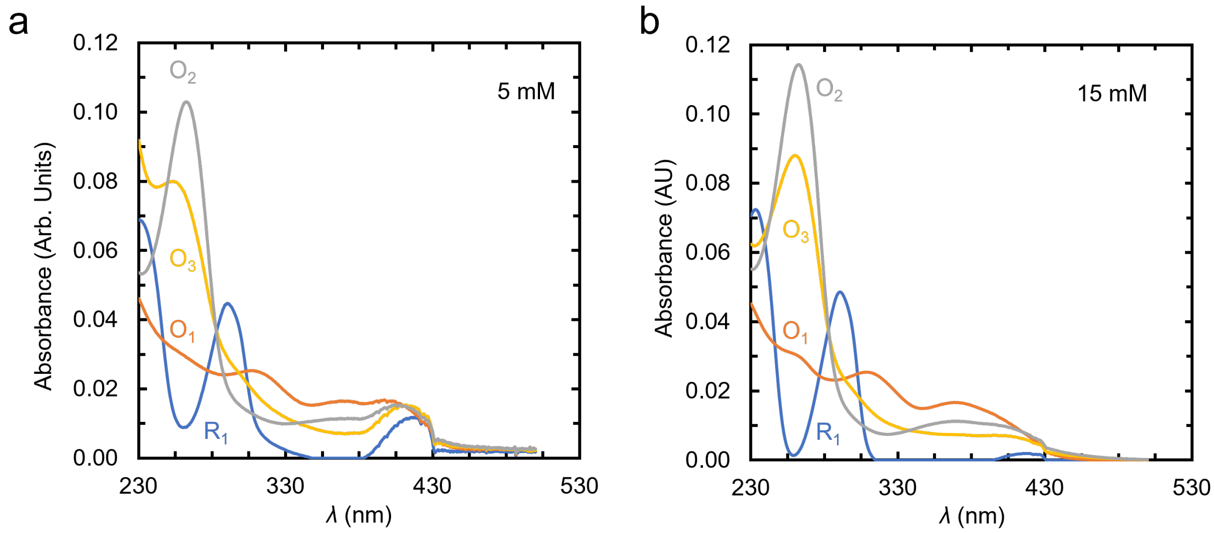

Supplementary Figure 12. Estimated spectra (using MCR-ALS) of BQDS derivatives for *operando* UV-vis experiments with BQDS concentrations of (a) 5 mM and (b) 15 mM.

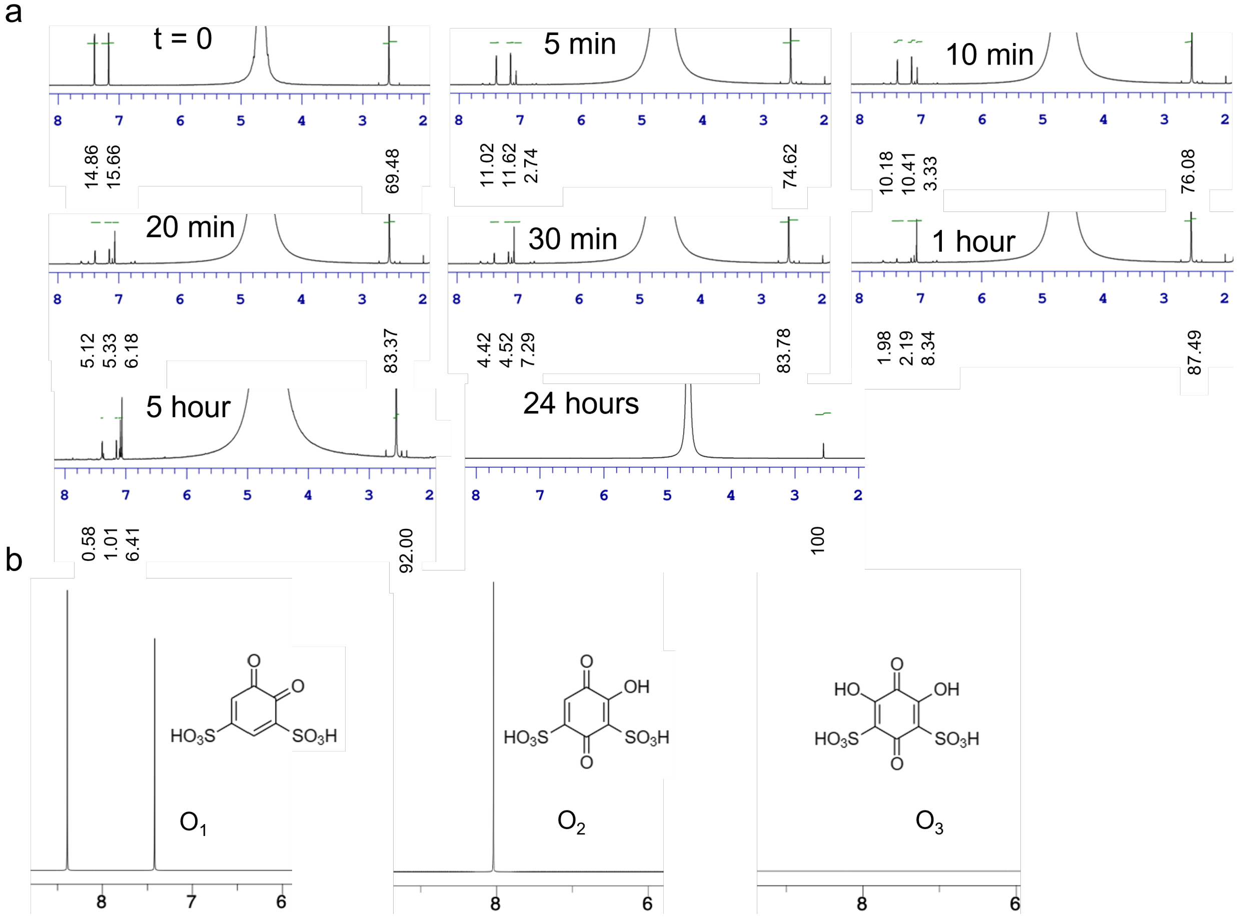

Supplementary Figure 13. (a) NMR scans of BQDS aliquots taken at various time intervals. (b) Simulated NMR spectra of O_1, O_2, and O_3. The peak at 2.6 ppm comes from 40 mM methanesulfonic acid used as the internal standard.

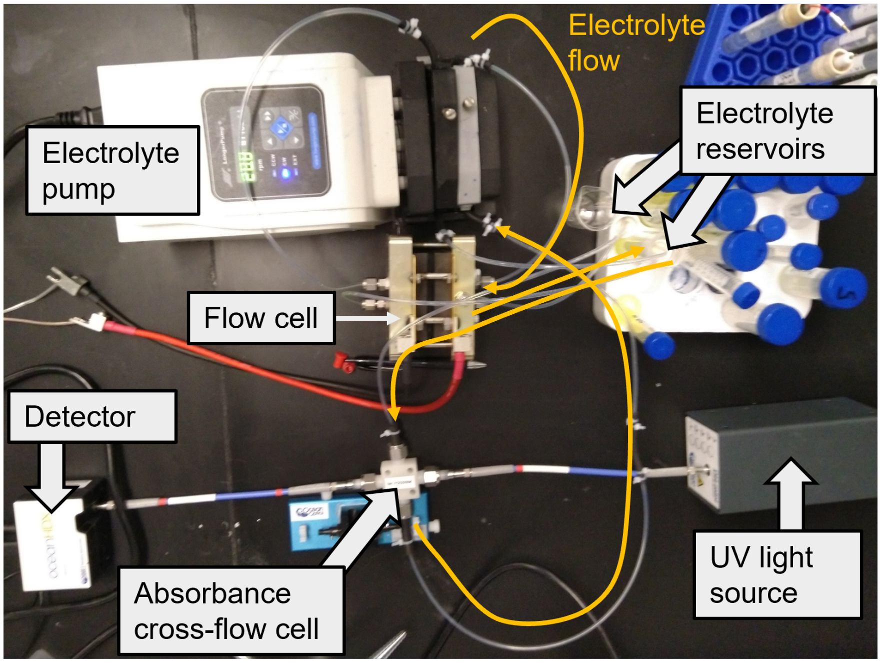

Supplementary Figure 14. Photograph of flow cell setup with absorbance cross-flow cell for *operando* UV-vis measurements. Yellow arrows depict the flow of electrolyte from the reservoir to the cross-flow cell, electrochemical flow cell, and electrolyte pump.

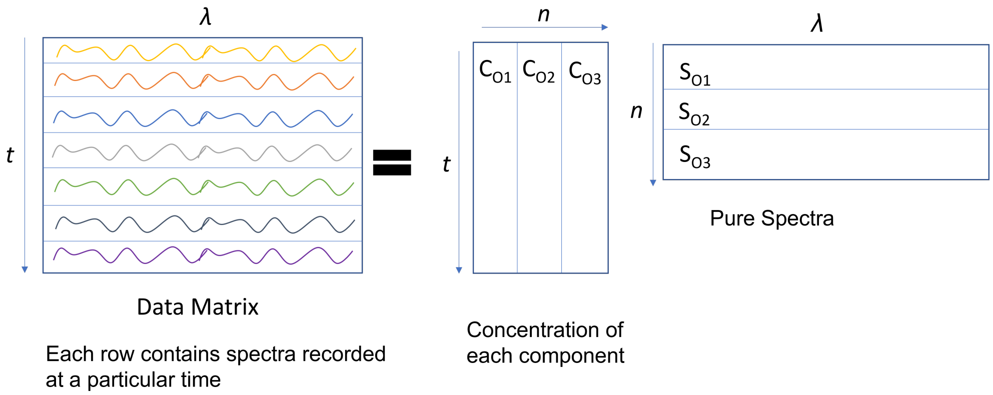

Supplementary Figure 15. Beer-Lambert law represented as a spectral data matrix which is the product of concentration and pure spectra matrices.
